# A digital health registry with clinical decision support for improving quality of antenatal care in Palestine (eRegQual): a pragmatic, cluster-randomised, controlled, superiority trial

**DOI:** 10.1016/S2589-7500(21)00269-7

**Published:** 2022-01-25

**Authors:** Mahima Venkateswaran, Buthaina Ghanem, Eatimad Abbas, Khadija Abu Khader, Itimad Abu Ward, Tamara Awwad, Mohammad Baniode, Michael James Frost, Taghreed Hijaz, Mervett Isbeih, Kjersti Mørkrid, Christopher J Rose, J Frederik Frøen

**Affiliations:** a Global Health Cluster, Division for Health Services, Norwegian Institute of Public Health, Oslo, Norway; b Centre for Intervention Science in Maternal and Child Health (CISMAC), Centre for International Health, Department of Global Health and Primary Care, University of Bergen, Bergen, Norway; c Palestinian National Institute of Public Health, Ramallah, Palestine; d Health Information Systems Programme, Department of Informatics, University of Oslo, Oslo, Norway

## Abstract

**Background:**

Health worker compliance with clinical guidelines is enhanced by digital clinical decision support at the point of care. The Palestinian public health system is implementing a digital maternal and child health eRegistry with clinical decision support. We aimed to compare the quality of antenatal care between clinics using the eRegistry and those using paper-based records.

**Methods:**

The eRegQual cluster-randomised controlled trial was done in primary health-care clinics offering routine antenatal care in the West Bank, Palestine. The intervention was the eRegistry with clinical decision support for antenatal care, implemented in District Health Information Systems 2 (DHIS2) Tracker software. 133 clinics forming 120 clusters were included and randomised; clusters were randomly assigned (1:1) to either the control (paper-based documentation) or intervention (eRegistry with clinical decision support) groups. The primary process outcomes were appropriate screening and management of anaemia, hypertension, and diabetes during pregnancy and foetal growth monitoring. The primary health outcome at delivery was a composite of moderate or severe anaemia; severe hypertension; large-for-gestational-age baby; malpresentation and small-for-gestational-age baby undetected before delivery. Data were analysed with mixed-effects logistic regression, accounting for clustering within clinics and pregnancies as appropriate. This trial is registered with the ISRCTN registry (ISRCTN18008445).

**Findings:**

Between Jan 15 and Sept 15, 2017, 3219 pregnant women received care in the intervention clinics (n=60 clusters) and 3148 pregnant women received care in the control primary health-care clinics (n=59 clusters). Compared with the control group, the intervention led to higher guideline adherence for screening and management of anaemia (1535 [28·9%] of 5320 *vs* 2297 [44·3%] of 5182; adjusted odds ratio [OR] 1·88 [95% CI 1·52–2·32]), hypertension (7555 [94·7%] of 7982 *vs* 7314 [96·6%] of 7569; adjusted OR 1·62 [95% CI 1·29–2·05]), and gestational diabetes (1726 (39·7%) of 4348 *vs* 2189 (50·7%) of 4321; adjusted OR 1·45 [95% CI 1·14–1·83]) at eligible antenatal contacts. Only 599 (9·4%) of 6367 women attended the full antenatal care schedule, and better care provision did not translate to fewer adverse health outcomes in the intervention clusters (700 cases; 21·7%) compared to the control clusters (688 cases; 21·9%; adjusted OR 0·99; 95% CI 0·87–1·12).

**Interpretation:**

Clinical decision support for antenatal care in the eRegistry was superior for most process outcomes but had no effect on the adverse health outcomes. The improvements in process outcomes strengthen the evidence for the WHO guideline for digital client tracking with clinical decision support in lower-middle-income settings. Digital health interventions to address gaps in attendance might help achieve effective coverage of antenatal care.

**Funding:**

European Research Council and Research Council of Norway.

**Translation:**

For the Arabic translation of the abstract see Supplementary Materials section.

## Introduction

Universal effective coverage of antenatal care is key to fulfil the promises of the UN's Global Strategy for Women's, Children's, and Adolescents’ Health, and to achieve the UN Sustainable Development Goal (SDG) targets for maternal and child health. The *Lancet Global Health* Commission on high-quality health systems in the SDG era asserted that “providing health services without guaranteeing a minimum level of quality is ineffective, wasteful, and unethical”.^1^ This remains a challenge in low-income and middle-income countries (LMICs).^1^, ^2^

Digital health is one of the accelerators that can support achievement of the health-related SDGs. Clinical decision support is among the most promising of digital health interventions in improving quality of care in LMICs,^3^ and the WHO guidelines for digital health interventions recommend individual-level digital records with clinical decision support. Yet, WHO underlines that, despite the promise of digital health interventions, evidence of their effectiveness is scarce, and there are multiple contextual implementation challenges.^4^

**Research in context**

**Evidence before this study**

We searched PubMed for systematic reviews of clinical decision support systems published between Jan 1, 2011, and March 15, 2021, with the search term “computerized decision support system” without any restrictions by language, health condition, or population. The initial search returned 63 systematic reviews. We excluded systematic reviews of implementation challenges or successes of clinical decision support systems (n=5), those in which computerised decision support systems were evaluated as part of a package of interventions (n=10), systematic reviews of only computerised provider order entries to reduce medication errors (n=8), systematic reviews of decision support delivered on paper (n=1), and primary studies and systematic reviews of non-health-sector interventions (n=3). Most randomised controlled trials included in the remaining 36 systematic reviews showed moderate improvements in processes of care and little or no improvements in clinical endpoints and mortality. The 2019 WHO guideline on digital interventions for health-systems strengthening endorses the implementation of clinical decision support combined with digital tracking of clients in low-income and middle-income settings. The systematic review that formed the basis of the WHO recommendation included five trials of effectiveness. The strength of evidence was reported as low to moderate, although the systematic review only included clinical decision support for health workers delivered on mobile devices. We did not identify any trials of clinical decision support for antenatal care in low-income and middle-income settings.

**Added value of this study**

To our knowledge, eRegQual is the first large cluster-randomised controlled trial to evaluate a system for digital tracking of clients with integrated clinical decision support in a lower-middle-income setting. The added value of this study is the demonstration of an improvement in the quality of antenatal care processes in primary health care. The pragmatic trial design, embedded in a large-scale implementation of a digital health information system in a setting with only paper records, sets a methodological example for generating evidence for digital health interventions for health-systems strengthening.

**Implications of all the available evidence**

The WHO recommendation comes with certain context-specific caveats, including the need to implement clinical decision support consisting of tasks that are feasible for the health-care worker to perform. The eRegistry is the routine health information system in Palestine and has been implemented at scale, a testament to the feasibility of longitudinal tracking of clients with embedded digital health interventions in lower-middle-income settings. In the eRegQual trial, we developed and tested the effectiveness of a clinical decision support system based on guidelines of care in the public health system. We found improvements in the screening and management of important conditions during pregnancy, showing the possible benefits of such a system to optimise health-system performance.

A synthesis of high-quality systematic reviews found that clinical decision support in general, across digital and manual versions, had moderate effects on clinical practice in most trials, and better patient outcomes only in a minority of cases.^5^ Digitising and automating clinical decision support have proven to be notable success factors.^6^ Among 32 randomised controlled trials, in which digital clinical decision support provided recommendations automatically at the point of care, 30 (94%) significantly improved clinical practice.^7^ In primary preventive care, such as antenatal care, most implementations of clinical decision support showed positive effects on clinical practice. However, few studies assessed digital clinical decision support, and often assessed these systems as stand-alone tools not integrated with electronic health records.^8^ The use of electronic health records in itself improves clinical guideline adherence,^9^ and integrating clinical decision support into electronic health records has a further moderate effect on reducing morbidity, although mortality reductions are yet to be shown in randomised controlled trials.^10^ To the best of our knowledge, none of the published systematic reviews has identified randomised controlled trials of clinical decision support integrated with electronic health records for antenatal care, or included any randomised controlled trials from LMICs.^8^, ^9^, ^10^

Despite the scarcity of evidence, maternal health is a domain for which digital applications with clinical decision support are commonly implemented in LMICs,^11^ but typically as stand-alone systems, and they are poorly documented and evaluated, and unavailable for widespread use.^12^, ^13^ WHO's 2017 mobile health report identified such multiplicity from a large variety of systems and lack of integration, standards, and evidence as barriers to global uptake of digital health interventions.^14^ In many LMICs, health-care providers already spend an estimated one-third of their time on repetitive documentation and reporting of data.^1^ This provides a further incentive to integrate digital health interventions within routine health information systems in LMICs, typically through the free, open-source District Health Information Systems 2 (DHIS2) platform.

We partnered with the Palestinian health authorities to implement a maternal and child health eRegistry for the public primary health-care system in the DHIS2 Tracker software.^15^ An eRegistry corresponds to the WHO definition of a digital health intervention with “longitudinal tracking of clients’ health status and services received” in electronic health records with integrated clinical decision support;^4^ the functionalities of the eRegistry evaluated here closely align with the newly released WHO digital adaptation kit for antenatal care.^16^ We designed the initial roll-out of the maternal and child health eRegistry as a cluster-randomised controlled trial. The maternal and child health eRegistry has since been implemented across the West Bank and Gaza Strip, replacing the paper-based records.

We aimed to estimate the effectiveness of the clinical decision support system in the maternal and child health eRegistry in improving the provision of timely and appropriate screening and management for important conditions in routine antenatal care, and health outcomes for mothers and newborn babies.

## Methods

### Study design and participants

This pragmatic, cluster-randomised, controlled, superiority trial was done in primary health-care clinics offering routine antenatal care in the West Bank, Palestine. The trial protocol with detailed methods and context has been published.^17^ In brief, the unit of randomisation was the public primary health-care clinic reporting to the Palestinian Ministry of Health. In the West Bank, there are about 400 public clinics providing routine antenatal care for low-risk pregnancies. Each clinic has a predefined referral clinic, and there are 86 referral clinics that provide care for pregnant women with certain risk factors or conditions. Private providers and the UN Relief and Works Agency for Palestine Refugees in the Near East (UNRWA) also offer antenatal care. About half of all pregnant women receive antenatal care in the public sector, and more than 80% deliver in government-run hospitals.

Implementation of the maternal and child health eRegistry started in 2014 and comprised five governorates (geographical and organisational districts of the health-care system) with 180 clinics. We excluded from the trial those clinics that were level 1 (small clinics that operate only once a week and typically enrol fewer than 10 pregnant women per year; n=5) or with no pregnant women enrolled in 2013 (n=5), those that were combined with a referral clinic at the same location (n=24), or those that were part of another simultaneous health-systems study addressing the quality of antenatal care (n=13).

Trial registration was initiated at the start of the trial on Jan 17, 2017, but the final version of the protocol was not submitted before March 28, 2017, because of ongoing discussions among the research team and stakeholders about postponing trial initiation to allow a longer run-in period. At the end, the January initiation date was kept, as per protocol, and the trial was retrospectively registered with the ISRCTN registry (ISRCTN18008445). We report findings in accordance with the CONSORT extension for cluster trials (appendix 2 p 2).^18^

### Randomisation

133 clinics forming 120 clusters were included and randomised; smaller clinics were grouped to form clusters of two or three clinics. Clusters were randomly assigned 1:1 to either the control (paper-based documentation) or intervention (eRegistry with clinical decision support) groups. A statistician at the Centre for Intervention Science in Maternal and Child Health (CISMAC), University of Bergen, Norway, who was not involved in other trial activities did the randomisation. Randomisation was stratified by governorate and constrained on the number of new enrolments of pregnancies per year (to reflect the size and thus clinic level and days of operation per week); laboratory availability (which could affect care health-care providers’ performance of screening tests); proportion of new enrolments of women older than 40 years (to reflect general health and risk status); and the proportion of primiparous women (to reflect risk status). The statistician first generated 10 000 randomisation sequences with Stata, version 14, after which the 1000 best balanced allocations for the predefined covariates were identified. One of these 1000 randomisation allocations was then randomly selected.

### Procedures

The intervention was the eRegistry's clinical decision support for antenatal care. The eRegistry is meant for use during client consultations, when health-care providers enter clinical data into digital client records, containing the same data as the paper-based records used before the introduction of the eRegistry. The paper-based records (control) and digital client records (intervention) include sociodemographic information, medical and obstetric history, clinical and ultrasound examinations, and laboratory results. The digital data entry triggers the clinical decision support based on the rules for screening and management from the antenatal care guidelines set by the Palestinian Ministry of Health (appendix 2 p 5). These guidelines require five antenatal care visits for low-risk women and have not been revised to follow the newer 2016 WHO guidelines for antenatal care.^19^ Full details of the intervention are provided in accordance with the mHealth Evaluation, Reporting and Assessment (mERA) guidelines (appendix 2 p 7).

Between June 1 and Oct 30, 2016, clinics assigned to the intervention were provided with desktop computers and internet connection. All health-care providers (n=327) in the intervention clinics—nurses, midwives, and doctors—received training on data entry, creating and retrieving digital client records, and interpreting the clinical decision support functionalities in the eRegistry. Each health-care provider was given a username and password to access the eRegistry via an internet browser. The digital health records follow up individual women and can be accessed by multiple health-care providers, and the system tracks which health-care provider accesses the files and what they enter. A link to a demo version of the eRegistry, where readers can try the digital data entry and clinical decision support functionalities, is available on request to the corresponding author.

No financial or other incentives were provided to the health-care providers. More than 50 maternal and child health supervisors, maternal and child health doctors, and nursing directors from all districts, in addition to relevant central-level staff, participated in a 2-day workshop done in Ramallah in January, 2016. This was followed by multiple 1-day training workshops to train all care providers in all districts. There were no changes to guidelines during the trial. Health-system supervisors carried out periodic supervision visits to all clinics in both trial groups. The eRegistry was and continues to be mandatory for use by health-care providers as the main clinical documentation tool. All paper records were removed from the intervention clinics during the trial period. Routines for replacing malfunctioning computers and providing a back-up wireless internet solution within 24 h in cases of internet disruptions, systems for monitoring use of the eRegistry, and periodic data quality checks are described in the standard operating procedures of the eRegistry in Palestine,^15^ and were applicable to the intervention clinics. All included clinics were notified by the Ministry of Health about the research. Client information posters of the ongoing research were supplied to all enrolled clinics.

Health-care providers in control clinics continued using paper-based files throughout the trial period, and received the eRegistry with clinical decision support after study completion in the second half of 2018.

### Outcomes

Outcome measures that reflect the overall quality of antenatal care were selected through national stakeholder and expert group consultations (appendix 2 p 8). The clinical decision support prompts health-care providers to undertake specific activities of screening and management and these activities correspond to our outcome definitions. Among more than 500 rules in the clinical decision support rules engine, the following sentinel rules were selected: the primary process (adherence to guidelines) outcomes of screening and management of anaemia, hypertension, and diabetes during pregnancy and foetal growth monitoring (table 1). For those with a normal screening test result, screening was considered complete. For those detected with a condition, follow-up of the screening test result with correct management provided a successful primary outcome. We only included antenatal care in the clinics included in the trial; antenatal care at a referral clinic was excluded from our analysis.

**Table 1:** Definitions of eligible antenatal contacts, screening tests, and management for the process (adherence) outcomes based on the guidelines for routine low-risk antenatal care in the West Bank, Palestine

	**Screening test**	**Management**		**Overall outcome definition**
		Eligible conditions	Management algorithm	
**Anaemia during pregnancy**				
First antenatal contact	Haemoglobin test	Mild anaemia (haemoglobin 10·0–10·9 g/dL)	Repeat haemoglobin test within 4 weeks*	Screening with haemoglobin test and screening normal; and appropriate management if anaemia is detected
Antenatal contact at 24–28 weeks	Haemoglobin test	Moderate anaemia (haemoglobin 7·0–9·9 g/dL)	Repeat haemoglobin test within 4 weeks*	Screening with haemoglobin test and screening normal; and appropriate management if anaemia is detected
Antenatal contact at 36 weeks	Haemoglobin test	Severe anaemia (haemoglobin <7·0 g/dL)	Referral to hospital	Screening with haemoglobin test and screening normal; and appropriate management if anaemia is detected
**Hypertension during pregnancy**				
First antenatal contact	Blood pressure measurement	Mild gestational hypertension (systolic blood pressure 140–149 mm Hg or diastolic blood pressure 90–99 mm Hg)	Repeat blood pressure measurement within 4 days of the first measurement	Screening with blood pressure measurement and blood pressure within the normal range; and appropriate management if gestational or chronic hypertension is detected
Antenatal contact at 16 weeks	Blood pressure measurement	Moderate gestational hypertension (systolic blood pressure 150–159 mm Hg or diastolic blood pressure 100–109 mm Hg)	Referral to high-risk clinic or hospital	Screening with blood pressure measurement and blood pressure within the normal range; and appropriate management if gestational or chronic hypertension is detected
Antenatal contact at 18–22 weeks	Blood pressure measurement	Severe gestational hypertension (systolic blood pressure >160 mm Hg or diastolic blood pressure >110 mm Hg)	Referral to high-risk clinic or hospital	Screening with blood pressure measurement and blood pressure within the normal range; and appropriate management if gestational or chronic hypertension is detected
Antenatal contact at 24–28 weeks	Blood pressure measurement	Chronic hypertension	Referral to high-risk clinic or hospital	Screening with blood pressure measurement and blood pressure within the normal range; and appropriate management if gestational or chronic hypertension is detected
Antenatal contact at 32 weeks	Blood pressure measurement	Chronic hypertension	Referral to high-risk clinic or hospital	Screening with blood pressure measurement and blood pressure within the normal range; and appropriate management if gestational or chronic hypertension is detected
Antenatal contact at 36 weeks	Blood pressure measurement	Chronic hypertension	Referral to high-risk clinic or hospital	Screening with blood pressure measurement and blood pressure within the normal range; and appropriate management if gestational or chronic hypertension is detected
**Diabetes during pregnancy**				
First antenatal contact before 24 weeks	Urine sugar test or blood sugar test	Positive random blood sugar test (≥140 mg/dL)	Referral to high-risk clinic or hospital	Screening with urine or blood sugar test and screening normal; and appropriate management if a high blood sugar is detected
First antenatal contact after 28 weeks	Blood sugar test	Positive random blood sugar test (≥140 mg/dL)	Referral to high-risk clinic or hospital	Screening with urine or blood sugar test and screening normal; and appropriate management if a high blood sugar is detected
Antenatal contact at 24–28 weeks	Blood sugar test	Positive random blood sugar test (≥140 mg/dL)	Referral to high-risk clinic or hospital	Screening with urine or blood sugar test and screening normal; and appropriate management if a high blood sugar is detected
**Abnormal foetal growth**				
First antenatal contact after 20 weeks	Symphysis fundal height or ultrasound examination	Discrepancy between fundal height and gestational age of greater than 2 or lesser than −2; ultrasound suspected foetal growth abnormalities	Ultrasound examination within 1 week; referral to high-risk clinic or hospital	Screening with antenatal ultrasound or symphysis fundus height measurement and screening normal; and appropriate management if a discrepancy between the symphysis fundus height and gestational age or foetal growth abnormalities are detected in ultrasound
**Malpresentation during pregnancy**†				
Antenatal contact at 36 weeks	Presentation checked by abdominal palpation or antenatal ultrasound	Non-cephalic presentation	Referral to hospital	Screening with abdominal palpation, or antenatal ultrasound for foetal presentation and presentation cephalic; and appropriate management if non-cephalic presentation

* Haemoglobin is measured after 4 weeks of treatment with oral iron and folic acid supplementation for mild and moderate anaemia. Treatment with oral iron and folic acid supplements was not measured as part of the management due to unreliable documentation in the clinical records.

† Secondary outcome.

The primary health outcomes, a composite of conditions at delivery that good-quality antenatal care aims to detect and prevent, were moderate or severe anaemia (haemoglobin at admission <10 g/dL); severe hypertension (systolic blood pressure ≥160 mm Hg or diastolic ≥110 mm Hg, or both, at admission); term large-for-gestational-age baby (≥90th percentile [birthweight of 3258 g] at 37 weeks of gestation);^20^ term small-for-gestational-age baby (≤10th percentile [birthweight of 2394 g] at 37 weeks of gestation)^20^ undetected during antenatal care; and malpresentation at delivery (non-cephalic presentation at or after 36 weeks of gestation) undetected during antenatal care.

The secondary outcomes were stillbirth (baby born with no signs of life at or after 28 weeks of gestation); women's antenatal care attendance from 16 weeks to 36 weeks according to guidelines; and screening and management of malpresentation (table 1). For antenatal care attendance, we calculated the proportion of women who attended each recommended antenatal care visit, among those registered for antenatal care before that visit and not sent to a referral clinic or hospital.

### Data collection and masking

We included data on all women attending the allocated clinics for antenatal care in the index pregnancy, with no eligibility criteria related to individual women's characteristics. Data for process (adherence) outcomes were obtained from the client records. Before recruitment, control clinics were supplied with records marked with serial numbers and instructed to use them sequentially. The records were collected after completion of pregnancy; we ensured that all numbered and completed records were returned. All data in the paper-based records were entered into the eRegistry every month of follow-up by four trained registry staff, to ensure all further data management (including evaluation of outcomes) would be identical for intervention and control data. Birth outcomes from public hospitals were entered by hospital staff into their routine health information system and exported every month and merged into the eRegistry with the unique identifiers of the mothers. Data on births in private hospitals and hospitals run by non-governmental organisations were collected by the Ministry of Health staff by use of a standardised form and merged into the eRegistry.

The data in the eRegistry belong to the Palestinian Ministry of Health. All data management procedures were handled in accordance with the ministry's legal framework and standard operating procedures for the eRegistry. For this trial, only predefined and anonymised data needed for the outcomes analyses were extracted. CISMAC, independently of the trial researchers and sponsors, conducted trial monitoring of recruitment readiness in September, 2016, and midway through monitoring in August, 2017, 7 months after recruitment started.

Neither the health-care providers nor the registry staff who digitised the paper-based records could be masked to the allocation due to the nature of the intervention. However, health-care providers and data collectors were masked to the outcome measures, and to minimise any bias in data collection the data collectors were trained to digitise the entire paper record, with multiple data points beyond trial outcomes. 370 paper records were digitised by two data collectors for consistency of data entry. Both hospital staff collecting pregnancy outcomes and trial statisticians were masked to group allocation. We analysed each primary outcome separately, using dummy randomisation variable codes generated by CISMAC (A and B for outcome 1, C and D for outcome 2, and so on). The codes were provided to the statistician as allocation groups (intervention and control for each set) after completion of analyses.

This health systems research uses health data derived from the eRegistry, anonymised at the source. Trial participants were clinics and their professional staff, but women attending the clinics did not fulfil the criteria of the Ottawa Statement for being individual research participants and so were not required to provide informed consent for the use of anonymised data from the eRegistry.^21^ Health-care providers had no meaningful opportunity to refuse the intervention during a national roll-out to their workplace and were subjected to minimal risk as data were derived at the anonymised cluster level. The Ministry of Health approved the study in their health system without obtaining individual consent from employees of their clinics. The study was approved by the Palestinian Health Research Council (PHRC/HC/04/14) and exempt from ethical review by the Regional Committee for Health Research Ethics - Section South East B, Norway (REK/2016/264 B). The final version of the study protocol was submitted to both ethics committees before recruitment commenced.

### Statistical analysis

We assumed a 5% significance level, a cluster size coefficient of variation of 0·85, and intracluster correlation coefficients of 0·01 for adverse pregnancy outcomes and 0·04 for the process outcomes. We used the clustersampsi^22^ command for Stata and calculated that 120 clusters, recruiting for 8 months, and a mean cluster size of 44 pregnancies, would provide more than 90% power to detect a 15–25% change in the overall primary process (adherence) outcomes of screening and management and a 25–30% change in the composite adverse pregnancy outcome.

We computed baseline characteristics of pregnant women at the individual level, in categories predefined by the study. We used mixed-effects logistic regression to estimate the relative odds of each process outcome under intervention versus control conditions, adjusting for clustering within clinic and pregnancy as appropriate. We plotted marginal predictive probabilities of antenatal care attendance and successful screening and management with respect to the variables used to restrict randomisation. Age was either incorrectly coded or missing for no more than 1–3% of women across the process outcomes, and we therefore did complete case analyses.^23^ We re-expressed adjusted odds ratio (OR) estimates as additional numbers of women per thousand who would be expected to be successfully screened and managed under intervention versus control conditions (baseline risks were imputed from control group odds).^24^

An adverse health outcome was defined to have occurred if at least one of the constituent outcomes occurred, and not to have occurred if none occurred. We used Little's tests^25^ of the null hypotheses that missing values of the constituent outcomes were jointly missing completely at random and covariate-dependent missing. We used multiple imputation via chained equations^26^ to create and analyse 50 datasets with multiple imputation, and combined estimates for each outcome using Rubin's rules (appendix 2 p 9).^27^ For comparison, we also did a complete case analysis under the missing completely at random assumption. We estimated the intracluster correlation coefficient using the complete cases.

We adjusted for the stratification variable^28^ as a fixed effect in all analyses except for severe hypertension, a constituent of the adverse health outcome (by chance, this outcome could be predicted perfectly by the stratification variable in a small proportion of imputed datasets). We also adjusted for variables used to constrain randomisation as fixed effects in all analyses, using individual-level rather than cluster-level measurements where possible.

We followed the intention-to-treat principle for all analyses: pregnant women were analysed in the groups to which the clinics were randomised and—except for the complete case analyses—all pregnant women were included in the analyses. We computed 95% CIs and used the significance criterion of p values less than 0·05 throughout. A per-protocol analysis was planned in case of severe disruptions in electricity or internet connection in some clusters, but this situation did not arise. Statistical analyses were done with Stata; version 14 was used for the power calculation and version 16 for data analysis.

### Protocol deviations

We have not presented data on blood pressure, haemoglobin, urine glucose test at booking visit, and foetal presentation as background characteristics since they are potentially dependent on the intervention. We used mixed-effects logistic regression instead of generalised estimating equations and therefore report cluster-specific OR estimates rather than marginal risk ratio estimates. We adjusted for stratification and constraining variables on the basis of guidance from the European Medicines Agency^28^ and research that was unavailable during protocol development. We used plots of marginal predictive probabilities rather than spider graphs.

### Role of the funding source

The funders of the study had no role in study design, data collection, data analysis, data interpretation, writing of the report, or the decision to submit the manuscript for publication.

## Results

**Figure 1:**
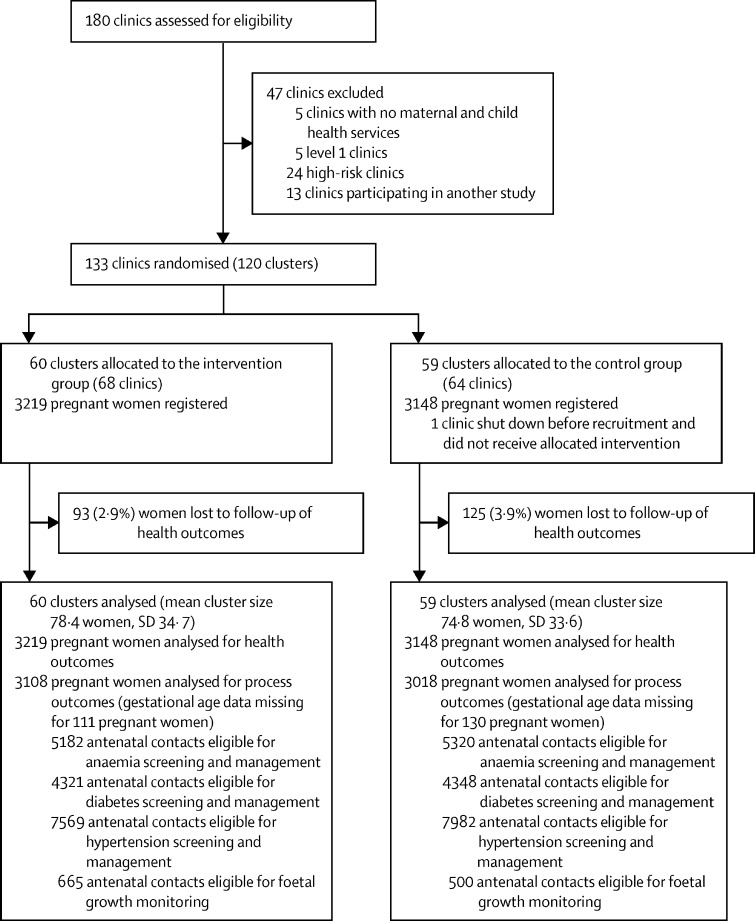
Trial profile

Between Jan 15 and Sept 15, 2017, 3219 pregnant women presented themselves for antenatal care in the intervention group (n=60 clusters, mean cluster size 78·4 pregnancies per cluster) as did 3148 pregnant women in the control group (n=59 clusters, mean cluster size 74·8; figure 1). All women completed antenatal care in the clinic they were registered at or in a referral clinic; there was no crossover between trial groups. Independent of this study, the Ministry of Health shut down one control clinic before the start of recruitment. We captured birth outcome data for all women except for 93 (2·9%) in the intervention group and 125 (3·9%) in the control group. The majority of deliveries took place in government-run hospitals (2297 [74·3%] in the intervention group and 2149 [72·2%] in the control group). Data on gestational age were not available in the records for 130 (4·1%) women in the control group and 111 (3·4%) women in the intervention group.

Laboratories were available in 30 intervention and 29 control clinics. In the intervention group, 1930 (59·9%) pregnant women received antenatal care in clinics with a laboratory versus 1735 (55·1%) in the control group. Ultrasound was available in 39 intervention and 40 control clinics. In the intervention group, 2475 (76·8%) pregnant women received antenatal care in clinics with ultrasound versus 2325 (73·8%) in the control group. Pregnant women's background characteristics were similar in the two groups (table 2), with a few exceptions: the control group included more primiparous women and documented more previous caesarean sections. Mean birthweight at term was 3257 g (95% CI 3244–3267) across both groups.

**Table 2:** Baseline characteristics of pregnant women

	**Control group (n=3148)**	**Intervention group (n=3219)**
**Maternal age (years)**		
≤20	442 (14·0%)	499 (15·5%)
21–25	1150 (36·5%)	1117 (34·7%)
26–30	828 (26·3%)	838 (26·0%)
31–35	400 (12·7%)	503 (15·6%)
36–40	226 (7·2%)	215 (6·7%)
>40	34 (1·1%)	46 (1·4%)
Data missing	68 (2·2%)	1 (0·0%)
**Parity**		
Primiparous women	740 (23·5%)	611 (19·0%)
**Average monthly household income (Israeli new shekel)**		
≤200	122 (3·9%)	93 (2·9%)
201–900	1659 (52·7%)	1678 (52·1%)
901–1824	1004 (31·9%)	970 (30·1%)
1825–3054	184 (5·8%)	201 (6·2%)
≥3055	27 (0·9%)	25 (0·8%)
Data missing	152 (4·8%)	252 (7·8%)
**Years of education**		
<10	324 (10·3%)	326 (10·1%)
10–13	1316 (41·8%)	1375 (42·7%)
>13	1403 (44·6%)	1420 (44·1%)
Data missing	105 (3·3%)	98 (3·0%)
**Age at first pregnancy (years)**		
≤20	1522 (48·3%)	1577 (49·0%)
21–25	1278 (40·6%)	1289 (40·0%)
26–30	226 (7·2%)	243 (7·5%)
31–35	41 (1·3%)	42 (1·3%)
36–40	18 (0·6%)	9 (0·3%)
>40	3 (0·1%)	3 (0·1%)
Data missing	60 (1·9%)	56 (1·7%)
**Medical and obstetric history**		
Type 2 diabetes	13 (0·4%)	15 (0·5%)
Caesarean section	330 (10·5%)	277 (8·6%)
Gestational diabetes	32 (1·0%)	25 (0·8%)
Perinatal death	58 (1·8%)	43 (1·3%)
Post-partum haemorrhage	81 (2·6%)	74 (2·3%)
Antepartum haemorrhage	44 (1·4%)	42 (1·3%)
Spontaneous miscarriage	155 (4·9%)	95 (3·0%)
Pre-eclampsia	20 (0·6%)	35 (1·1%)
**Body-mass index (kg/m^2^)**		
<18·5	108 (3·4%)	101 (3·1%)
18·5–24·9	1149 (36·5%)	1279 (39·7%)
25·0–29·9	819 (26·0%)	985 (30·6%)
≥30·0	485 (15·4%)	554 (17·2%)
Data missing	587 (18·6%)	300 (9·3%)

Data are n (%).

Women were more often appropriately screened for risk factors and referred to high-risk clinics in the intervention group (565 [17·6%] of 3219) than in the control group (397 [12·6%] of 2751), resulting in fewer low-risk women (n=2654) for routine antenatal care following the registration of the pregnancy in the intervention group than in the control group (n=2751).

Despite follow-up rates of 96·6% (6149 of 6367) for birth outcomes, a considerable proportion of birth outcome data were not documented in hospital health information systems. In the control group, data were missing for gestational age at delivery in 235 (7·4%) women, for birthweight in 327 (10·3%) women, for haemoglobin at admission to delivery in 868 (27·5%) women, and for blood pressure at admission to delivery in 961 (30·5%) women. In the intervention group, data were missing for gestational age at delivery in 231 (7·1%) women, for birthweight in 300 (9·3%) women, for haemoglobin at admission to delivery in 685 (21·2%) women, and for blood pressure at admission to delivery in 1139 (35·3%) women. For process outcomes, lack of documentation in client records was interpreted as non-performance of screening or management, and there were no missing data.

Women were more often appropriately screened and managed for anaemia, gestational diabetes, and hypertension in the intervention group than in the control group, with adjusted ORs ranging from 1·45 to 1·88 (table 3).

**Table 3:** Effect of eRegistry's clinical decision support on process outcomes of screening and management of conditions during antenatal care and health outcomes at delivery

	Control group*(n=3148)	Intervention group*(n=3219)	**Adjusted odds ratio (95% CI)**†
**Anaemia**			
Screening and management during eligible antenatal contacts	1535/5320 (28·8%)	2297/5182 (44·3%)	1·88 (1·52–2·32)
Screening during eligible contacts	1434/4513 (31·7%)	2159/4407 (48·9%)	..
Management of anaemia	101/807 (12·5%)	138/775 (17·8%)	..
**Diabetes**			
Screening and management during eligible antenatal contacts	1726/4348 (39·7%)	2189/4321 (50·7%)	1·45 (1·14–1·83)
Screening during eligible contacts	1714/4318 (39·6%)	2182/4279 (50·9%)	..
Management of diabetes	12/30 (40%)	7/42 (17%)	..
**Hypertension**			
Screening and management during eligible antenatal contacts	7555/7982 (94·7%)	7314/7569 (96·6%)	1·62 (1·29–2·05)
Screening during eligible contacts	7536/7884 (95·6%)	7266/7470 (97·3%)	..
Management of hypertension	19/98 (19·4%)	48/99 (48·5%)	..
**Abnormal foetal growth**			
Screening and management during eligible antenatal contacts	394/500 (78·8%)	458/665 (68·9%)	0·59 (0·37–0·96)
Screening during eligible contacts	386/467 (82·6%)	450/610 (73·7%)	..
Management of abnormal foetal growth	8/33 (24·2%)	8/55 (14·5%)	..

Data are n/N (%), unless otherwise stated.

* Unadjusted.

† Adjusted for clustering and for repeated antenatal care visits by a woman.

Anaemia was screened for, and managed as appropriate, in 28·8% of eligible antenatal care contacts in the control group versus 44·3% of eligible antenatal care contacts in the intervention group (table 3), corresponding to 97 (95% CI 60–138) additional women per 1000 expected to be successfully screened with the intervention compared to control conditions. In the control group, screening detected 807 cases of mild or moderate anaemia, of which 101 (12·5%) were correctly managed, whereas 775 cases were detected by screening in the intervention group, of which 138 (17·8%) were correctly managed. Only two cases of severe anaemia were documented, both in the control group.

Gestational diabetes was successfully screened for and managed in 39·7% of eligible antenatal care contacts in the control group versus 50·7% in the intervention group (table 3), corresponding to approximately 41 (95% CI 13–73) additional women per 1000 expected to be successfully screened with the intervention compared to control conditions. The finding appears to be mostly driven by better screening in the intervention group (50·9%) compared to the control group (39·6%; table 3). Few cases of high blood sugar were detected in both groups: 30 in the control group, of which 12 were correctly managed with a referral; and 42 in the intervention group, of which seven were correctly managed with a referral.

Hypertension screening and management had an overall high baseline value, with successful screening and management in 94·7% of eligible antenatal care contacts in the control group and 96·6% of eligible antenatal care contacts in the intervention group, corresponding to approximately 11 (95% CI 6–15) additional women per 1000 expected to be successfully screened with the intervention compared to control conditions. Management of hypertension was better in the intervention group, with 48 (48·5%) of 99 women referred to a higher health facility or monitored in the same clinic as appropriate, compared to 19 (19·4%) of 98 women in the control group (table 3).

Appropriate screening and management for abnormal foetal growth was recorded more often in the control group than in the intervention group (table 3). This was therefore the only primary process outcome for which there was no improvement in the intervention group, with 394 (78·8%) of 500 eligible antenatal visits successfully screened and managed in the control group compared with 458 (68·9%) of 665 in the intervention group. Both screening and management alike had higher unadjusted percentages in the control versus the intervention group (table 3).

**Figure 2:**
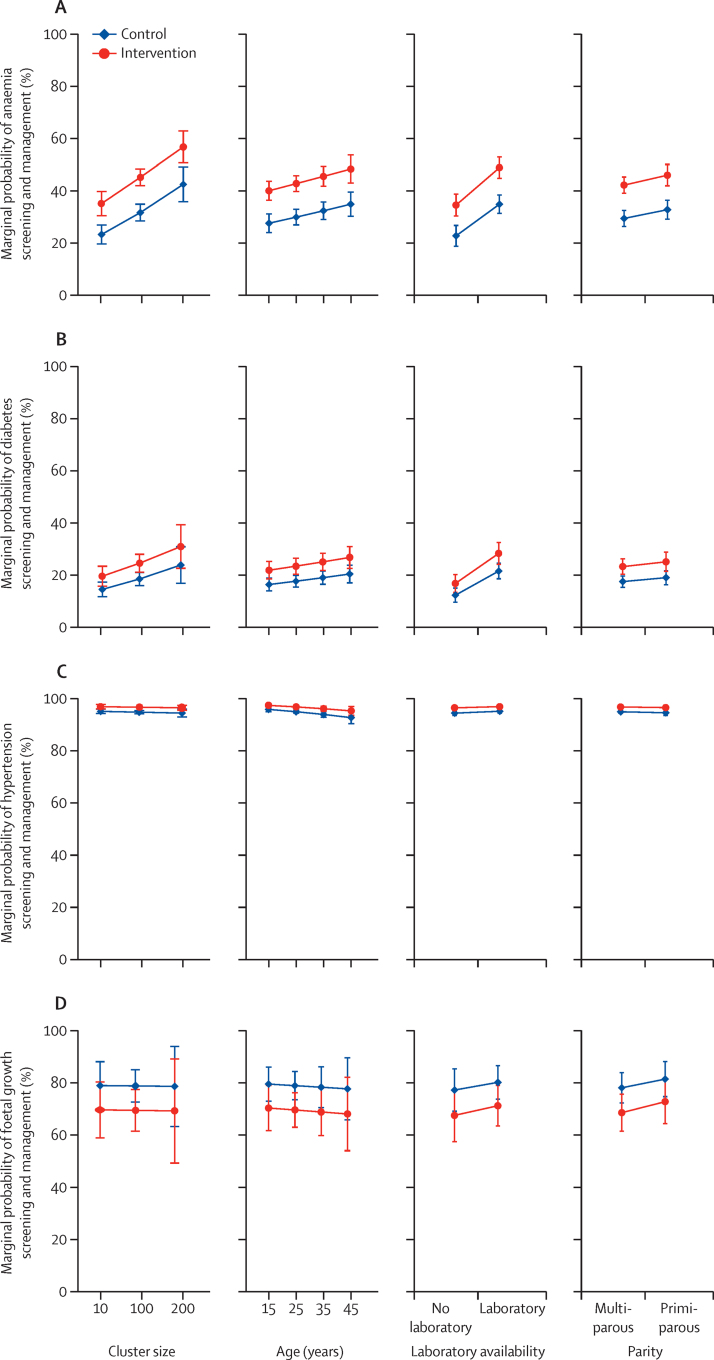
Plots showing marginal probability of the primary process (adherence) outcomes with respect to cluster size, age of the woman, laboratory availability, and parity (A) Anaemia screening and management. (B) Diabetes screening and management. (C) Hypertension screening and management. (D) Foetal growth screening and management.

Irrespective of the intervention, we estimate the probability of successful screening and management of anaemia and diabetes to be higher in large clinics (≥200 annual pregnancy registrations), clinics with a laboratory, and among primiparous and older women (≥40 years; figure 2). The probability of screening and management of hypertension and abnormal foetal growth was not associated with any of the above factors.

Outcome data were missing for 33·8% (2153 of 6367) of the composite outcome. We were unable to reject the missing completely at random (p=0·15) and covariate-dependent missing hypotheses (p=0·64). Distributions of the original and the first five imputed datasets are shown in appendix 2 (p 9). We found no difference in adverse pregnancy outcomes between the control (21·9%) and intervention groups (21·7%; table 4). The intracluster correlation coefficient was estimated to be close to zero and no greater than 0·007 (upper bound of 95% CI).

**Table 4:** Effect of eRegistry's clinical decision support on health outcomes at delivery

	Control group*(n=3148)	Intervention group*(n=3219)	**Adjusted odds ratio**†**(95% CI)**
Adverse pregnancy outcome	688 (21·9%)	700 (21·7%)	0·99 (0·87–1·12)
Moderate or severe anaemia	44 (1·4%)	37 (1·1%)	0·82 (0·51–1·31)
Severe hypertension	16 (0·5%)	10 (0·3%)	0·61 (0·27–1·36)
Large-for-gestational-age baby	343 (10·9%)	384 (11·9%)	1·06 (0·90–1·25)
Small-for-gestational-age baby, undetected before delivery	226 (7·2%)	220 (6·8%)	1·00 (0·81–1·24)
Malpresentation at delivery, undetected before delivery	90 (2·9%)	84 (2·6%)	0·93 (0·68–1·27)

Data are n (%), unless otherwise indicated.

* Unadjusted.

† Adjusted for clustering, multiply imputed result.

Attendance at eligible antenatal care contacts was similar in the intervention and control groups (43% *vs* 44%; 4502 of 10475 opportunities for attendance *vs* 4912 of 11238 opportunities for attendance; appendix 2 p 15). Attendance was associated with clinic size (adjusted OR 1·53; 95% CI 1·18–2·00), but not laboratory availability (0·99; 0·78–1·27), age of the woman (1·01; 1·00–1·02), or parity (1·01; 0·89–1·15; appendix 2 p 16). Across the two groups, 599 (9·4%) of 6367 pregnant women attended the full schedule of routine antenatal care, following pregnancy registration (unadjusted: 323 [10·3%] of 3219 pregnant women in the control group and 276 [8·6%] of 3148 pregnant women in the intervention group).

Malpresentation was successfully screened and managed in 790 (77·5%) of 1021 eligible antenatal care contacts in the control group and 733 (80·2%) of 914 eligible antenatal care contacts in the intervention group (adjusted OR 1·42; 95% CI 0·92–2·19), thus showing no difference between the groups. There was no difference in stillbirth rates, with 20 stillbirths (six per 1000) in the control group, and 21 stillbirths (seven per 1000) in the intervention group (adjusted OR 1·07; 95% CI 0·57–2·00; appendix 2 p 15).

## Discussion

The eRegQual cluster-randomised controlled trial was done in primary health-care clinics providing antenatal care in a lower-middle-income setting. This pragmatic trial made use of an ongoing implementation as an opportunity to study a new health-systems approach. We found that a clinical decision support system for antenatal care improved screening and management of anaemia, diabetes, and hypertension. The intervention did not have an effect on foetal growth monitoring, antenatal care attendance, or adverse health outcomes at delivery.

A systematic review of 28 randomised controlled trials assessing the impact of clinical decision support found that the intervention had no effect on mortality, and a weak effect on morbidity, potentially mediated by improved adherence to clinical decision support recommendations.^10^ A systematic review from 2020 included 122 trials of clinical decision support, and reported absolute improvements of 5·8% for process outcomes, and a much smaller improvement in clinical endpoints.^29^ Similar findings are reflected in our trial. Randomised controlled trials have typically compared electronic health records with clinical decision support versus electronic health records without clinical decision support or paper-based records, or combinations of both. We compared the eRegistry's clinical decision support with only paper-based documentations and were unable to identify other trials with this design.

Foetal growth monitoring was the only primary process outcome for which the intervention did not improve quality of care. Our post-hoc exploration to validate this unexpected finding indicates that it is based on a documentation bias due to the design and unanticipated use of paper records in the control clinics. Fundal height screening on the paper records is documented on a table with gestational ages in one column, and fundal height in a corresponding row. However, some health-care providers inserted gestational age in the rows, leaving the gestational age column empty. This made the data appear to be a documented fundal height measurement, while it might have been a documented gestational age. In the eRegistry, however, gestational age was automatically calculated and only an entry for fundal height was available.

The adverse health outcomes in this trial can be affected by the quality of antenatal care. Most essential interventions in antenatal care rely on continuity of care (eg, repeated measures of blood pressure and haemoglobin levels). The effect of quality of care on pregnancy outcomes, therefore, depends on the utilisation of care, such that better effective coverage of essential interventions is achieved.^30^ Only 599 (9·4%) of 6367 women attended all routine antenatal care visits following pregnancy registration. The lack of effect on the health outcomes of this trial must be interpreted with such low attendance in mind. Utilisation of care should be addressed through further research and targeted interventions.

For countries considering infrastructure investments to transition from paper-based records to digital systems, replacing paper with “paper on screen” without integrating supporting tools for health-care providers represents an obsolete option. The eRegistry's clinical decision support is built in DHIS2, which is free for all to customise and use. For implementation of clinical decision support, adaptation to the local context by reconciling global standards of care with local clinical guidelines and work processes is crucial to improve acceptability and adherence. The process outcomes on which the intervention had an effect had clear clinical guidelines in the West Bank, that were well aligned with local practices.

The study had several limitations, including the potential documentation bias for foetal growth, as discussed above. Missing data for health outcomes was another limitation, but we addressed this issue using established statistical methods. The similar amount of missing data between the two groups and almost identical levels of adverse outcomes indicates that improved quality of care cannot achieve health improvements when the majority of women do not attend care. In conclusion, this trial responds to WHO's call for evidence of the effectiveness of guideline implementation based on digital tools,^16^ and showed that an eRegistry with clinical decision support for antenatal care implemented at scale in the West Bank resulted in improved screening and management, with no effect on adverse health outcomes at delivery or antenatal care attendance, compared to paper-based records.

## Data sharing

All data in the eRegistry belong to the Ministry of Health, Palestine, and will not be made available with this publication. However, the syntax for extraction of the exact dataset used in the trial analysis and a data dictionary is stored at the Palestinian National Institute of Public Health. The anonymised dataset used in this trial will be available for up to 5 years from the date of this publication. Requests for data access can be submitted to the Palestinian National Institute of Public Health at info@pniph.org. Anonymised, individual-level data along with a data dictionary will be provided, on condition that the requester submits a study protocol with clearly defined objectives, a predefined list of data points, and appropriate ethics approvals. The analysis code is publicly available at GitHub (multinormal/fhi.eRegQual.2020). The most recent version available at the time of writing is archived at Zenodo (https://doi.org/10.5281/zenodo.5557071).

## Declaration of interests

All authors were funded by grants from the European Research Council (Consolidator Grant, grant number 617639) and the Research Council of Norway (Globvac Grant, grant number 234376; and National Center of Research Excellence Grant, grant number 223269). The authors also declare non-financial support from the Centre for Intervention Science in Maternal and Child Health (CISMAC), University of Bergen, Norway.

## Supplementary Material

Arabic translation of the abstract
