# Supplementary material for: A digital health registry with clinical decision support for improving quality of antenatal care in Palestine (eRegQual): a pragmatic, cluster-randomised, controlled, superiority trial
Source: Lancet Digit Health. 2022 Jan 25;4(2):e126–36. doi: 10.1016/S2589-7500(21)00269-7 (PMC8811715; doi:10.1016/S2589-7500(21)00269-7)
Supplement: Arabic translation of the abstract [file mmc1.pdf]

THE LANCET
Digital Health

Supplementary appendix 1

This appendix formed part of the original submission and has been peer reviewed. We post it as supplied by the authors.

تم تقديم هذه الترجمة باللغة العربية من قبل المؤلفين ونعيد إنتاجها كما هو مُقدم. إنها لم تخضع لاستعراض الأقران. تم تطبيق عمليات تحرير لانسيت فقط على النص الأصلي باللغة الإنجليزية، والذي يجب أن يكون بمثابة مرجع لهذه المخطوطة.

This translation in Arabic was submitted by the authors and we reproduce it as supplied. It has not been peer reviewed. *The Lancet*'s editorial processes have only been applied to the original in English, which should serve as reference for this manuscript.

Supplement to: Venkateswaran M, Ghanem B, Abbas E, et al. A digital health registry with clinical decision support for improving quality of antenatal care in Palestine (eRegQual): a pragmatic, cluster-randomised, controlled, superiority trial. *Lancet Digit Health* 2022; **4:** e126–36.

السجل الإلكتروني لتحسين جودة رعاية الحوامل في فلسطين: دراسة عنقودية عشوائية

المقدمة:

تقوم رعاية الصحة الاولية في وزارة الصحة الفلسطينية بتطبيق سجل إلكتروني لصحة الأم والطفل مع دعم القرارمن أجل تعزيز امتثال الطاقم الصحي للإرشادات السريرية. لقد تم مقارنة جودة الرعاية المقدمة أثناء الحمل ما بين العيادات التي تستخدم السجل الإلكتروني وتلك التي تستخدم السجلات الورقية.

أساليب البحث:

أجريت هذه التجربة العنقودية العشوائية في عيادات الرعاية الصحية الأولية التي تقدم رعاية الحوامل الروتينية في الضفة الغربية- فلسطين. كان التدخل المستخدم في هذه الدراسة هو السجل الإلكتروني مع دعم القرار السريري، والذي تم تنفيذه باستخدام نظام المعلومات الصحي الكتروني للتتبع المناطقي (DHIS2). لقد تم تضمين 133 عيادة مكونة من 120 مجموعة بشكل عشوائي. تم تعيين العيادات بشكل عشوائي (1: 1) إما لمجموعة التحكم (التوثيق الورقي) أو التدخل (السجل الإلكتروني مع دعم القرار السريري). لقد تم دراسة التحسن في عملية تقديم الخدمات الصحية المتعلقة بالتقصي وإدارة الحالة في الرعاية أثناء الحمل وتضمنت الفحص المناسب والتدبير العلاجي لفقر الدم وارتفاع ضغط الدم والسكري أثناء الحمل ومراقبة نمو الجنين.

وكذلك دراسة النتائج الصحية الأولية عند الولادة: وتتكون من فقر الدم المتوسط أو الشديد، ارتفاع ضغط الدم الشديد، سوء وضعية الجنين بالرحم وكبر او صغر حجم الجنين بالنسبة لعمر الحمل والذي لم يتم اكتشافه قبل الولادة. لقد تم تحليل المعطيات باستخدام الانحدار اللوجستي المختلط الآثار، مع مراعاة حجم التجمعات وعدد حالات الحمل داخل العيادات. تم تسجيل هذه التجربة في سجل ISRCTN18008445 .

النتائج:

من الفتره بين 15 يناير 2017 إلى 15 سبتمبر 2017، تلقت 3219 سيدة الرعاية أثناء الحمل من عيادات الرعاية الصحية الأولية التي تم تطبيق التدخل فيها (عدد = 60) وتلقت 3148 سيدة الرعاية أثناء الحمل من عيادات الرعاية الصحية الأولية التي لم يتم تطبيق التدخل فيها (ن = 59 مجموعة). مقارنة بمجموعة التحكم لقد أدى التدخل إلى زيادة الإلتزام بفحوصات وإدارة حالات فقر الدم بنسبة (1535

[28.9%] من 5320, مقابل 2297 [44.3%] من 5182, بنسبة بنسبة فردية (OR)1.88[95% فاصل ثقة (CI) 1.52 – 2.32]),

ارتفاع الضغط (7555 [94.7%] من 7982 بمقابل 7314 [96.6%] من 7569، بنسبة فردية (OR) 1.62[95% CI 1.29-2.05]) ، سكري الحمل بنسبة (1726[39.7%] من 4348 بمقابل 2189 [50.7%] من 4321، بنسبة فردية (OR) 1.45[95% CI 1.14-1.83]) في زيارات رعاية الحمل. فقط 599 (9.4%) من 6367 النساء أتممن برنامج زيارات رعاية الحمل بشكل كامل وإن تقديم رعاية أفضل لم ينعكس على بعض مخرجات الصحة في عيادات التدخل (700 حالة، 21.7%) مقارنة بـعيادات عدم التدخل (688 حالة، 21.9%) (بنسبة فردية 0.99 [95% فاصل ثقة (CI) 0.87 – 1.12])

تفسير النتائج:

لقد كان دعم القرار السريري للرعاية أثناء الحمل باستخدام التسجيل الإلكتروني ذا تأثير إيجابي على نتائج عملية تقديم الخدمات الصحية المتعلقة بالتقصي وإدارة الحالة في الرعاية أثناء الحمل ولكن لم يكن له تأثير سلبي على النتائج الصحية.

إن التحسن الملاحظ في مؤشرات تقديم الرعاية يدعم إرشادات منظمة الصحة العالمية المتعلقة بالتدخلات الصحية الرقمية مع دعم القرار السريري في المناطق ذات الدخل المتوسط والمنخفض. إن استخدام الأنظمة الرقمية لتحسين حضور السيدات لرعاية الحوامل ممكن أن يساعد في تحقيق تغطية فعالة للرعاية أثناء الحمل.
