# Supplementary material for: A digital health registry with clinical decision support for improving quality of antenatal care in Palestine (eRegQual): a pragmatic, cluster-randomised, controlled, superiority trial
Source: Lancet Digit Health. 2022 Jan 25;4(2):e126–36. doi: 10.1016/S2589-7500(21)00269-7 (PMC8811715; doi:10.1016/S2589-7500(21)00269-7)
Supplement: Supplementary appendix [file mmc2.pdf]

THE LANCET
Digital Health

Supplementary appendix 2

This appendix formed part of the original submission and has been peer reviewed. We post it as supplied by the authors.

Supplement to: Venkateswaran M, Ghanem B, Abbas E, et al. A digital health registry with clinical decision support for improving quality of antenatal care in Palestine (eRegQual): a pragmatic, cluster-randomised, controlled, superiority trial. *Lancet Digit Health* 2022; **4:** e126–36.

Table of contents

CONSORT 2010 checklist of information to include when reporting a cluster randomised trial 2

Intervention: eRegistry with clinical decision support 5

Screenshots 5

Figure 1: Illustration of the eRegistry with clinical decision support showing the automated generation of a risk condition – mild anemia (right panel) – based on the entered hemoglobin value of 10 g/dl 5

Figure 2: Illustration of the eRegistry with clinical decision support showing the list of actions to be performed by the care provider during the given antenatal care visit 6

Figure 3: Illustration of the eRegistry with clinical decision support showing the automated generation of a risk condition – chronic hypertension (right panel) – based on the entered values of 150 mmHg systolic and 95 mmHg diastolic blood pressure 6

mHealth evidence reporting and assessment (mERA) checklist 7

National stakeholder consultation group and expert committee for the design and implementation of the eRegistry in the West Bank 8

Primary adverse health outcome - imputation 9

Secondary outcomes 15

Table. Effect of eRegistry's clinical decision support on secondary outcomes 15

Figure: Plot showing marginal probability of attendance at antenatal care (secondary outcome) with respect to cluster size, age of the woman, laboratory availability and parity 16

CONSORT 2010 checklist of information to include when reporting a cluster randomised trial

Section/Topic	Item No	Standard Checklist item	Extension for cluster designs	Page No
Title and abstract				
	1a	Identification as a randomised trial in the title	Identification as a cluster randomised trial in the title	**1**
	1b	Structured summary of trial design, methods, results, and conclusions (for specific guidance see CONSORT for abstracts)[1,2]	See table 2	**2**
Introduction				
Background and objectives	2a	Scientific background and explanation of rationale	Rationale for using a cluster design	**3, 4**
	2b	Specific objectives or hypotheses	Whether objectives pertain to the cluster level, the individual participant level or both	**4**
Methods				
Trial design	3a	Description of trial design (such as parallel, factorial) including allocation ratio	Definition of cluster and description of how the design features apply to the clusters	**5**
	3b	Important changes to methods after trial commencement (such as eligibility criteria), with reasons		**11**
Participants	4a	Eligibility criteria for participants	Eligibility criteria for clusters	**5**
	4b	Settings and locations where the data were collected		**5**
Interventions	5	The interventions for each group with sufficient details to allow replication, including how and when they were actually administered	Whether interventions pertain to the cluster level, the individual participant level or both	**6, 7**
Outcomes	6a	Completely defined pre-specified primary and secondary outcome measures, including how and when they were assessed	Whether outcome measures pertain to the cluster level, the individual participant level or both	**7, 8**
	6b	Any changes to trial outcomes after the trial commenced, with reasons		**Not applicable**
Sample size	7a	How sample size was determined	Method of calculation, number of clusters(s) (and whether equal or unequal cluster sizes are assumed), cluster size, a coefficient of intracluster correlation (ICC or *k*), and an indication of its uncertainty	**9**

	7b	When applicable, explanation of any interim analyses and stopping guidelines		**Not applicable**
Randomisation:				
Sequence generation	8a	Method used to generate the random allocation sequence		**5**
	8b	Type of randomisation; details of any restriction (such as blocking and block size)	Details of stratification or matching if used	**5**
Allocation concealment mechanism	9	Mechanism used to implement the random allocation sequence (such as sequentially numbered containers), describing any steps taken to conceal the sequence until interventions were assigned	Specification that allocation was based on clusters rather than individuals and whether allocation concealment (if any) was at the cluster level, the individual participant level or both	**9**
Implementation	10	Who generated the random allocation sequence, who enrolled participants, and who assigned participants to interventions	Replace by 10a, 10b and 10c	
	10a		Who generated the random allocation sequence, who enrolled clusters, and who assigned clusters to interventions	**5, 8, 9**
	10b		Mechanism by which individual participants were included in clusters for the purposes of the trial (such as complete enumeration, random sampling)	**8**
	10c		From whom consent was sought (representatives of the cluster, or individual cluster members, or both), and whether consent was sought before or after randomisation	**9**
Blinding	11a	If done, who was blinded after assignment to interventions (for example, participants, care providers, those assessing outcomes) and how		**9**
	11b	If relevant, description of the similarity of interventions		**Not applicable**
Statistical methods	12a	Statistical methods used to compare groups for primary and secondary outcomes	How clustering was taken into account	**9**
	12b	Methods for additional analyses, such as subgroup analyses and adjusted analyses		**10**
Results				

Participant flow (a diagram is strongly recommended)	13a	For each group, the numbers of participants who were randomly assigned, received intended treatment, and were analysed for the primary outcome	For each group, the numbers of clusters that were randomly assigned, received intended treatment, and were analysed for the primary outcome	**11**
	13b	For each group, losses and exclusions after randomisation, together with reasons	For each group, losses and exclusions for both clusters and individual cluster members	**11**
Recruitment	14a	Dates defining the periods of recruitment and follow-up		**11**
	14b	Why the trial ended or was stopped		**Not applicable**
Baseline data	15	A table showing baseline demographic and clinical characteristics for each group	Baseline characteristics for the individual and cluster levels as applicable for each group	**12**
Numbers analysed	16	For each group, number of participants (denominator) included in each analysis and whether the analysis was by original assigned groups	For each group, number of clusters included in each analysis	**11**
Outcomes and estimation	17a	For each primary and secondary outcome, results for each group, and the estimated effect size and its precision (such as 95% confidence interval)	Results at the individual or cluster level as applicable and a coefficient of intracluster correlation (ICC or k) for each primary outcome	**12-14**
	17b	For binary outcomes, presentation of both absolute and relative effect sizes is recommended		
Ancillary analyses	18	Results of any other analyses performed, including subgroup analyses and adjusted analyses, distinguishing pre-specified from exploratory		**14, 15**
Harms	19	All important harms or unintended effects in each group (for specific guidance see CONSORT for harms)[3]		**Not applicable**
Discussion				
Limitations	20	Trial limitations, addressing sources of potential bias, imprecision, and, if relevant, multiplicity of analyses		**16, 17**
Generalisability	21	Generalisability (external validity, applicability) of the trial findings	Generalisability to clusters and/or individual participants (as relevant)	**16, 17**
Interpretation	22	Interpretation consistent with results, balancing benefits and		**16, 17**

		harms, and considering other relevant evidence		
Other information				
Registration	23	Registration number and name of trial registry		**5**
Protocol	24	Where the full trial protocol can be accessed, if available		**5**
Funding	25	Sources of funding and other support (such as supply of drugs), role of funders		**11**

Intervention: eRegistry with clinical decision support

Screenshots

Figures 1 – 3 are screenshots of the MCH eRegistry as seen by the health care providers, using dummy client data to provide examples of the clinical decision support in the clinical records.

Figure 1: Illustration of the eRegistry with clinical decision support showing the automated generation of a risk condition – mild anemia (right panel) – based on the entered hemoglobin value of 10 g/dl

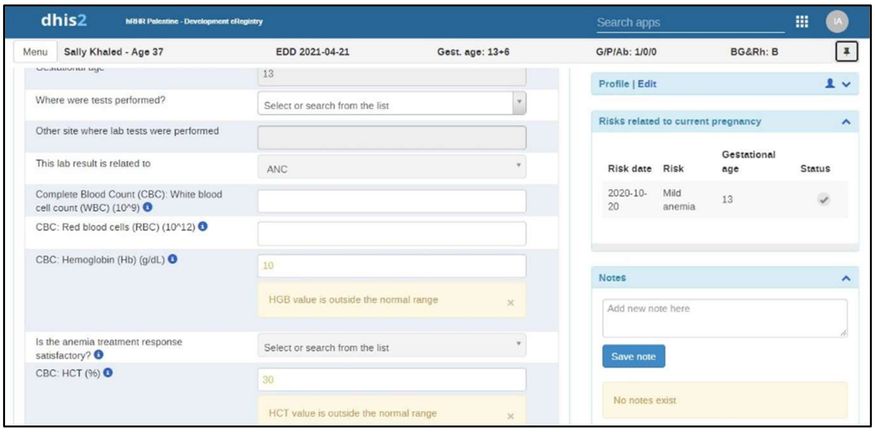

The top line and right-hand column on the screen show the care provider key information about the woman. Both are “pinned” and stay visible while the care provider scrolls through the checklist during ANC. In the top line, this includes her estimated day of delivery and current gestational age, computed from the last menstrual period date, or first trimester ultrasound, if done, as entered by the care provider during the booking visit. In this example, the care provider enters “10” as the result of a hemoglobin test, and the system responds by 1) a soft validation to minimize erroneous data entries, indicating that the value is outside the normal range, and 2) it identifies a hemoglobin value of 10 g/dL as mild anemia according to the Palestinian guidelines for ANC, and adds this information to the right-hand column as a “Risk related to the current pregnancy”. See also Figure 2.

Figure 2: Illustration of the eRegistry with clinical decision support showing the list of actions to be performed by the care provider during the given antenatal care visit

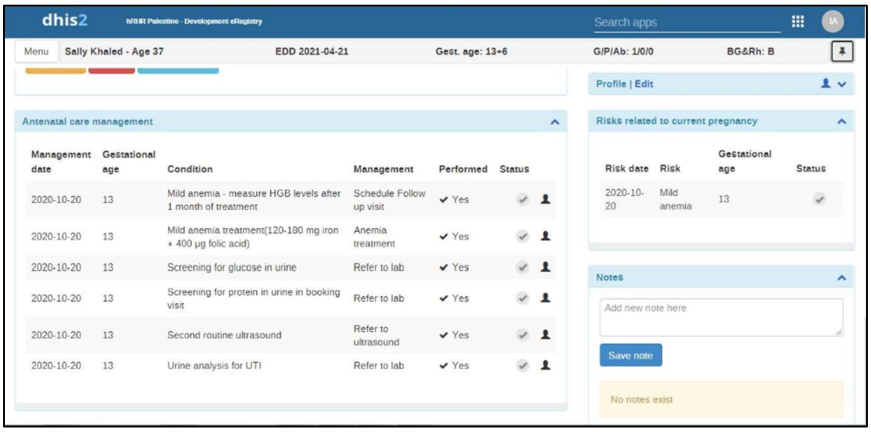

This screenshot is a continuation of the dummy client in Figure 1. At the end of all examinations and results, when the care provider reaches the bottom of the scrolling checklist, a summary management plan is presented. This includes the specific management for any complications identified, in this example treatment of mild anemia and re-testing of hemoglobin according to the Palestinian guidelines. It also lists the routine tests that the woman should be referred to in preparation for her next ANC visit, individualized to her gestational age and the guidelines. The care provider is expected to check off that the recommended management plan has been followed, or if not, enter a note on why not. This is, however, not a required field needed to continue using the system. See also Figure 3.

Figure 3: Illustration of the eRegistry with clinical decision support showing the automated generation of a risk condition – chronic hypertension (right panel) – based on the entered values of 150 mmHg systolic and 95 mmHg diastolic blood pressure

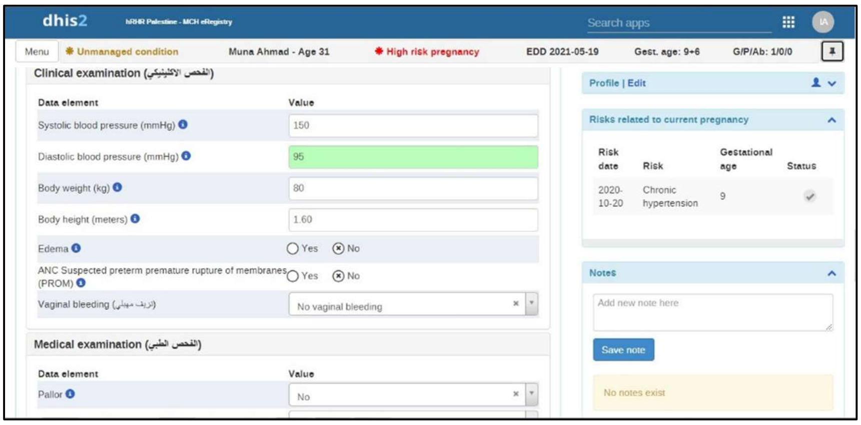

In this example of another dummy client, the care provider has entered a high diastolic blood pressure. The system combines the information on blood pressure with the computed gestational age (9 weeks + 6 days), and identifies that this represents chronic hypertension according to the guidelines. In the Palestinian guidelines, chronic hypertension in pregnancy falls under their definitions of a high-risk pregnancy that should be referred from the primary health clinic to a "High risk clinic". The system therefore flags this status as a high-risk pregnancy in the top line of key information about the pregnancy. As the care provider has not yet reached the end of the consultation and the recommended management plan, the woman has not yet been referred to the high-risk clinic. The top line therefore also flags that this woman has an unmanaged condition. This remains in the key information line during this and all future visits until the woman has been referred in compliance with the guidelines.

mHealth evidence reporting and assessment (mERA) checklist

Criteria	Description
Infrastructure	The eRegistry has been implemented a national level in primary healthcare in the West Bank, for use by healthcare providers of maternal and child. Most primary healthcare clinics in the West Bank have landline internet connection and one or two desktop computers, depending on the size of the clinic and number of staff. The Ministry of Health runs a central server center, where the national health information system data are stored.
Technology platform	The eRegistry is built in the free and open source DHIS2 Tracker. DHIS2 Tracker is designed to capture and manage individual-level data such as clinical records, either with the DHIS2 core software or with the DHIS2 Android capture app. In the West Bank, the eRegistry in DHIS2 Tracker is accessed through an internet browser. More details on DHIS2 Tracker can be found here: https://dhis2.org/tracker/
Interoperability/health information system (HIS) context	There are no other digital data collection systems in public primary healthcare for maternal and child health. Apart from the eRegistry for maternal and child health care, DHIS2 is also used within family practice in primary healthcare. All governmental hospitals in the West Bank use a proprietary software, not linked to the eRegistry. Private sector providers use their own digital or paper-based documentation systems.
Intervention delivery	Care providers in governmental primary healthcare clinics – nurses, midwives, non-nurse/midwife health workers, doctors with training in maternal and child health, specialists in obstetrics and gynecology – use the eRegistry to create and maintain digital client records of pregnant and postpartum women and newborn babies. A group of care providers and health system supervisors were selected and trained to be so-called super-users, to provide day to day support to the care providers. In the first phase of the implementation, 327 care providers received initial training over 3 days, followed by on-site support from the implementation team. The Palestinian National Institute of Public Health, the Norwegian Institute of Public Health, and the Ministry of Health, Palestine collaborated to implement the eRegistry. University of Oslo was responsible for software development and customization.
Intervention content	eRegistry's clinical decision support is based on the maternal and child health guidelines set by the Palestinian Ministry of Health. Prior to the customization of the eRegistry, the guidelines were discussed in detail in a national expert committee and a stakeholder group (details on page 2, 3). The guidelines and the content of the eRegistry are specific to the local context and have not been updated in accordance with the 2016 WHO guidelines for a positive pregnancy experience.
Usability/content testing	Co-design and development of eRegistry's clinical decision support largely followed the Principles of Digital Development*. The clinical guidelines for screening and management for specific conditions during pregnancy were discussed with the stakeholders. Workflow mapping exercises in primary healthcare were done to aid the design of the digital data entry and clinical decision support. Over a period of 6-8 months, the implementation team conducted field visits to gather user feedback on the data entry interface, and workflow and clinical decision support.
User feedback	In a user survey conducted in 2017, A majority (57%) reported the system to be easy to use and were satisfied with the tool (80%). Most respondents (59%) reported that the clinical decision support was somewhat easy or very easy. On the question "How often are you able to carry out the management as recommended by the MCH eRegistry?" 81% reported always or often, while 15% reported sometimes and only 4% seldom.
Access of individual participants	Clients accessing antenatal, postnatal, and newborn care at governmental primary healthcare clinics are part of the eRegistry; their clinical data are exclusively entered in eRegistry's digital client records.
Cost assessment	The items to be considered for cost assessment include hardware (in this study desktop computers and a dedicated server in the Palestinian National server-park); internet connection of 3G speed or higher (in this study fiber landlines to the majority of clinics); staff for the installation and configuration of DHIS2 Tracker with users; staff for training, supervising and providing support to users; staff for maintaining, updating and managing data in DHIS2 Tracker.

	More detailed guidance on cost assessments and planning is provided in the DHIS2 Tracker implementation guidance maintained by DHIS2.org here: https://docs.dhis2.org/en/implement/implement.html
Adoption inputs/program entry	Intervention implementation and maintenance is Ministry of Health-driven. Use of the eRegistry is mandatory for the care providers. All paper-based records were removed from the clinics and compulsory transition from paper. We engaged with health system supervisors, who work closely with nurses and midwives in the clinics, to ensure smooth transition to the eRegistry.
Limitations for delivery at scale	DHIS2 is the most commonly used national health information system in low- and middle-income countries, with nation-wide implementation in over 60 countries. Instances of DHIS2 Tracker is in use in over 80 countries, with over 30 countries reaching national scale during the COVID-19 pandemic for use in COVID-19 surveillance and immunization programs. Limitations for delivery at scale are largely restricted to costs, capacity, and pre-existing infrastructure, not the DHIS2 system. However, the browser-based implementation in this study requires internet access at scale, and the current Android version of DHIS2 Tracker needed for mobile connectivity does not support all functionalities used in this study.
Contextual adaptability	Since the eRegistry is built on the DHIS2 Tracker, it can be adapted to any context that uses the Tracker in DHIS2 core software for individual-level data collection. The clinical decision support functionality will need to be modified based on local clinical guidelines and workflow.
Replicability	A demo version of the eRegistry in Palestine, where users can log in and test, can be provided on request (also see screenshots on page 2, 3).
Data security	All data were collected in compliance with the legal framework and policies of the Palestinian Ministry of Health, as documented in the Data Security, Privacy and Confidentiality subsection of the Standard Operation Procedures for Routine Registry Operations - Implementation, Establishment and Maintenance of Mother and Child Health (MCH) Registry (2017). Direct access to personally identifiable data was controlled using strict role-based authority, assigned following existing permission levels within the health data access framework of the health system in Palestine. All persons involved with data entry, access or management provided confidentiality consent. Data for the study were hosted within the national health data centers for the West Bank and Gaza according to national protocols and managed by named personnel according to the Ministry of Health framework for the MCH eRegistry. DHIS2 adheres to OWASP recommendations for ensuring privacy and security and was configured to match national security requirements for data entry, access, storage, and transmission. The eRegistry in Palestine can only be accessed within the Ministry of Health secured VPN network, combined with the Kaspersky Security Center services, antivirus software with endpoint protections, and a Cisco ASA firewall to support IPS and threat management detection. More information about DHIS2 security can be found at https://dhis2.org/security/.
Compliance with national guidelines and regulatory statutes	Our intervention was implemented on a national scale in collaboration with the Ministry of Health and the Palestinian National Institute of Public Health to ensure full compliance with national guidelines and regulations.
Fidelity of the intervention	Since first implementation in 2016, the eRegistry has been used by all governmental primary healthcare clinics for antenatal, postnatal, and newborn care, without long-standing interruptions. Paper back-up files used during power outages are always computerized later, such that all women have a digital client record.

*Principles for Digital Development, available from: https://digitalprinciples.org/about/

National stakeholder consultation group and expert committee for the design and implementation of the eRegistry in the West Bank

Stakeholder consultation group	Number
Specialists - obstetrics and gynecology	4
Doctors with training in maternal and child health	15
Nursing (health system) supervisors	10

Nursing directors, Ministry of Health, Palestine	10
Representatives from the World Health Organization, office for West Bank and Gaza (oPT)	5

Expert committee	Number
Specialists - obstetrics and gynecology	4
Nursing directors, Ministry of Health, Palestine	1
Director of women's health department, Ministry of Health, Palestine	1
Head of community health department, Ministry of Health, Palestine	1
Midwives/ Nurses at primary healthcare clinics	3
Representatives from the World Health Organization, office for West Bank and Gaza (oPT)	4

Primary adverse health outcome - imputation

We imputed using the auxiliary variables trial arm, years of education, average monthly household income (log transformed due its skewed distribution), body mass index, ultrasound availability, and the variables used as constraints in the randomisation (cluster size, age, lab availability, and parity). We were not able to include auxiliary variables that indicated previous pregnancy with pre-eclampsia or previous history of GDM due to collinearity. We evaluated convergence of the imputation algorithm by inspecting trace plots and evaluated imputed data by inspecting kernel density estimates and histograms comparing the distributions of imputed and complete case data. The following figures show the distributions of the original and a selection of the imputed data.

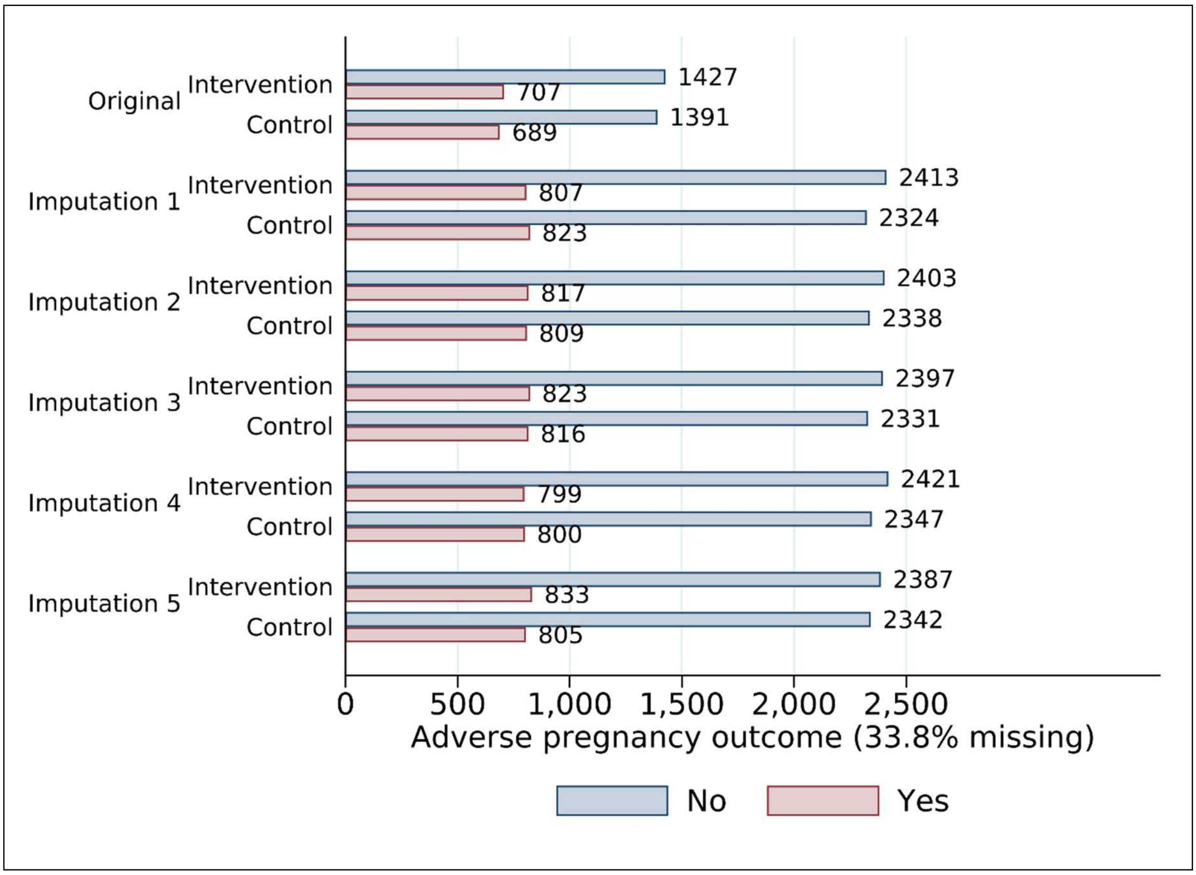
Original
Intervention
1427
707
Control
1391
689
Imputation 1
Intervention
2413
807
Control
2324
823
Imputation 2
Intervention
2403
817
Control
2338
809
Imputation 3
Intervention
2397
823
Control
2331
816
Imputation 4
Intervention
2421
799
Control
2347
800
Imputation 5
Intervention
2387
833
Control
2342
805
0
500
1,000
1,500
2,000
2,500
Adverse pregnancy outcome (33.8% missing)
No
Yes

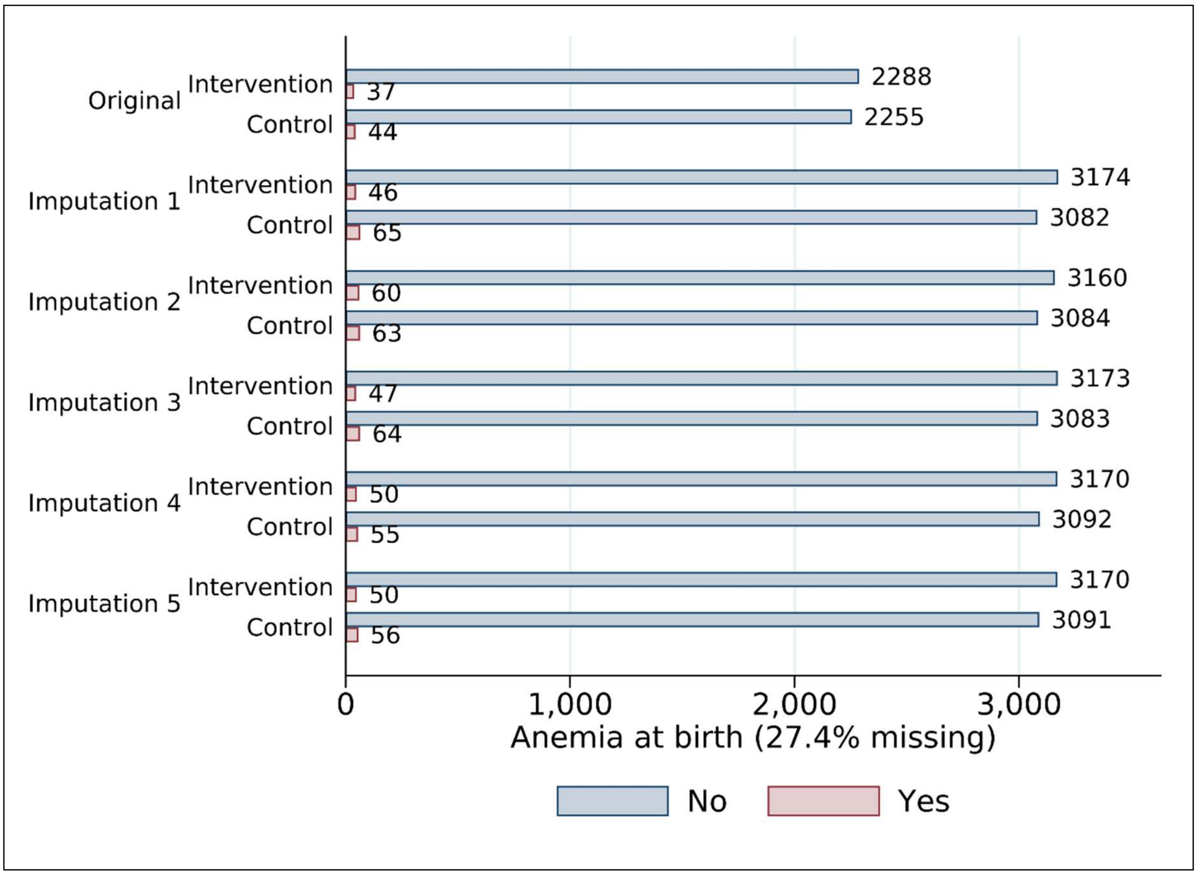
Original
Intervention
2288
37
Control
2255
44
Imputation 1
Intervention
3174
46
Control
3082
65
Imputation 2
Intervention
3160
60
Control
3084
63
Imputation 3
Intervention
3173
47
Control
3083
64
Imputation 4
Intervention
3170
50
Control
3092
55
Imputation 5
Intervention
3170
50
Control
3091
56
0
1,000
2,000
3,000
Anemia at birth (27.4% missing)
No
Yes

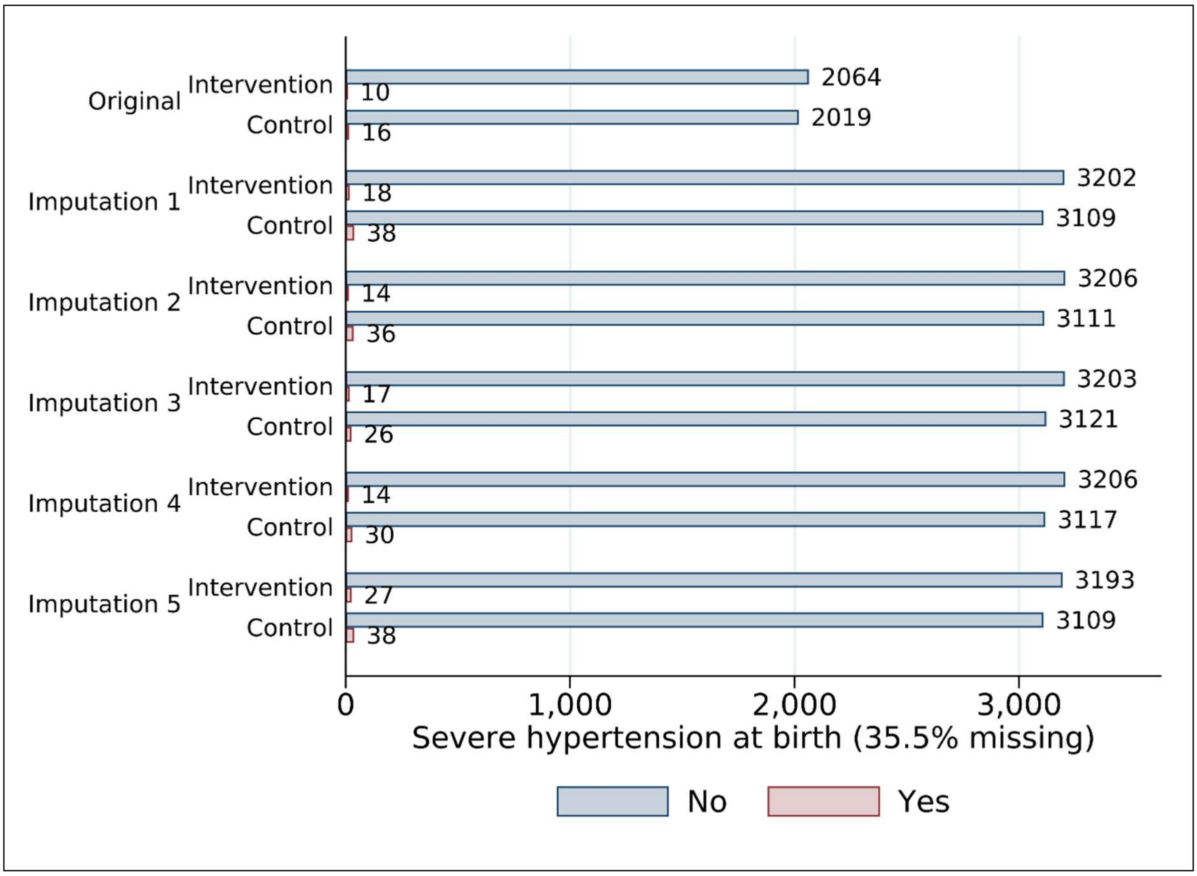
Original
Intervention
Control
Imputation 1
Imputation 2
Imputation 3
Imputation 4
Imputation 5
2064
10
2019
16
3202
18
3109
38
3206
14
3111
36
3203
17
3121
26
3206
14
3117
30
3193
27
3109
38
0
1,000
2,000
3,000
Severe hypertension at birth (35.5% missing)
No
Yes

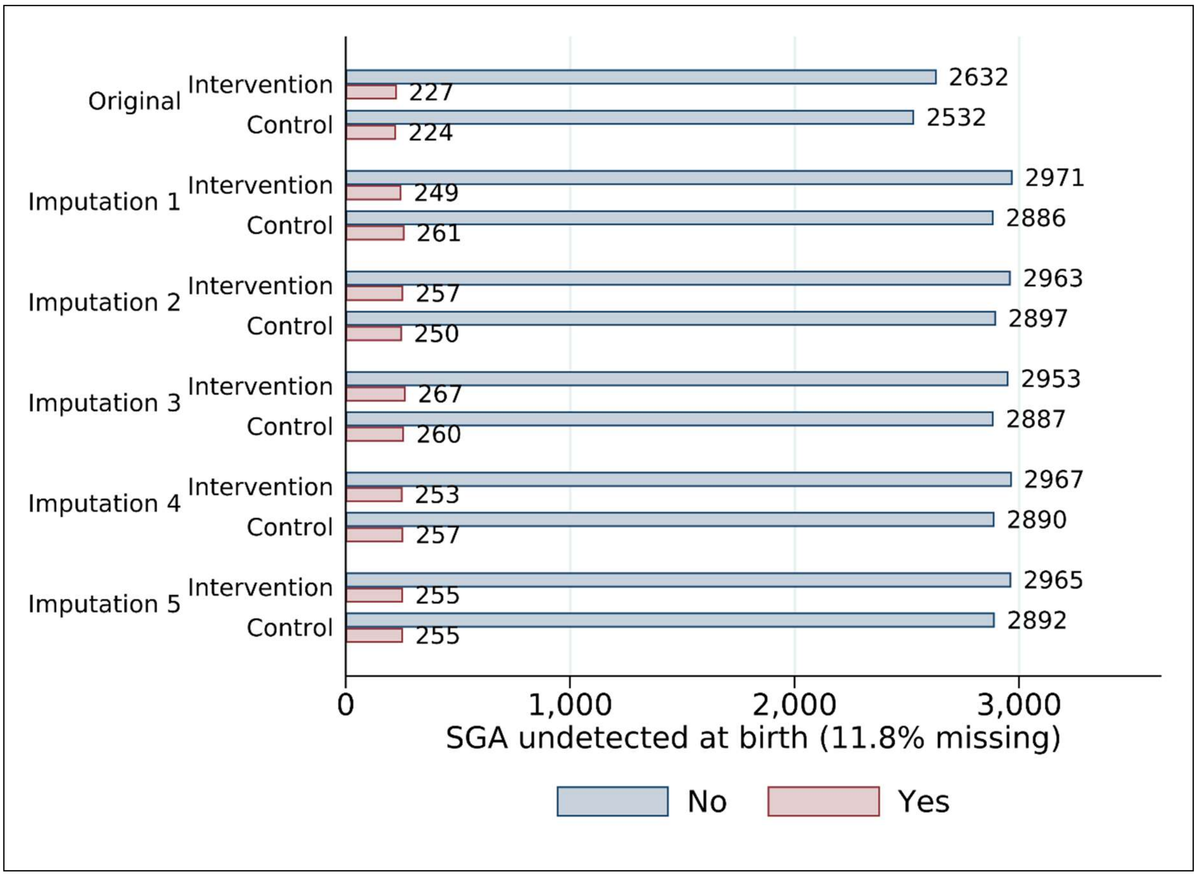
Original
Intervention
Control
Imputation 1
Imputation 2
Imputation 3
Imputation 4
Imputation 5
2632
227
2532
224
2971
249
2886
261
2963
257
2897
250
2953
267
2887
260
2967
253
2890
257
2965
255
2892
255
0
1,000
2,000
3,000
SGA undetected at birth (11.8% missing)
No
Yes

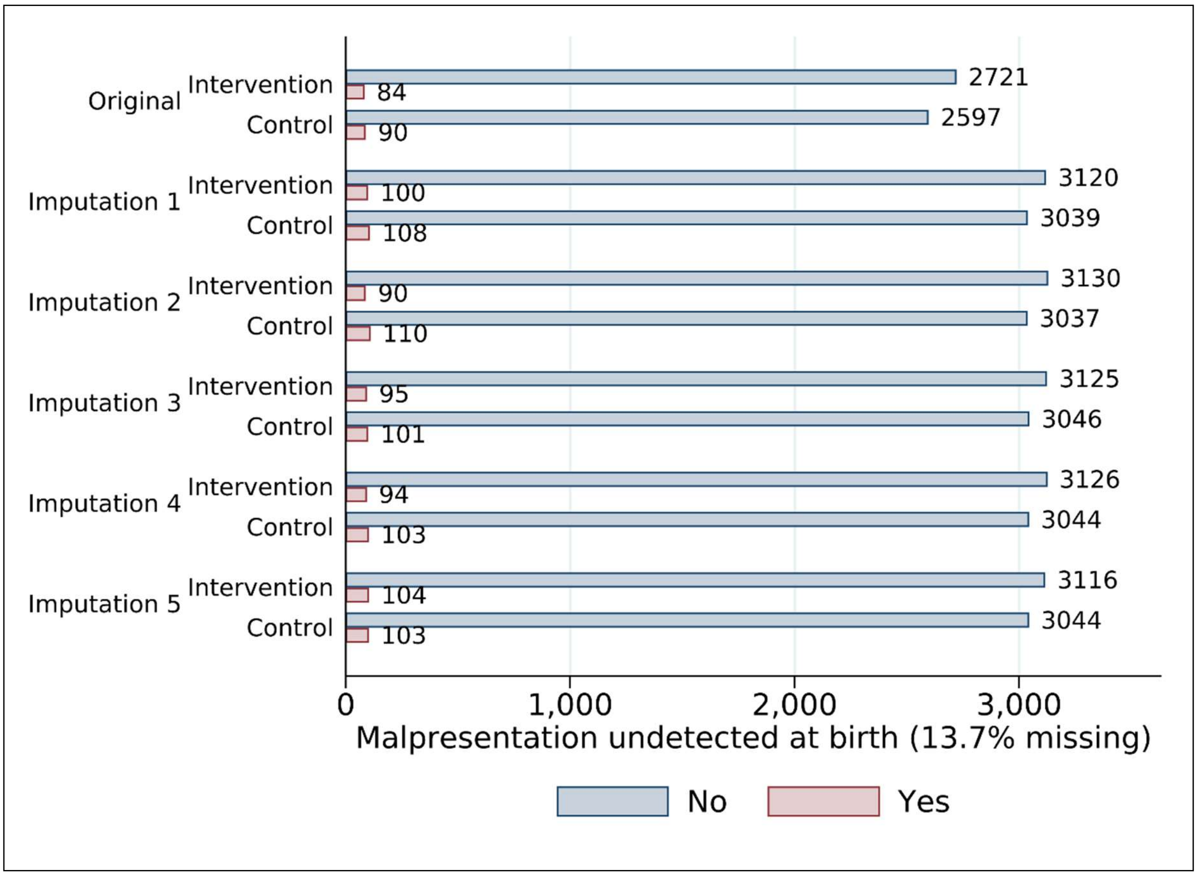
Original
Intervention
Control
Imputation 1
Imputation 2
Imputation 3
Imputation 4
Imputation 5
2721
84
2597
90
3120
100
3039
108
3130
90
3037
110
3125
95
3046
101
3126
94
3044
103
3116
104
3044
103
0
1,000
2,000
3,000
Malpresentation undetected at birth (13.7% missing)
No
Yes

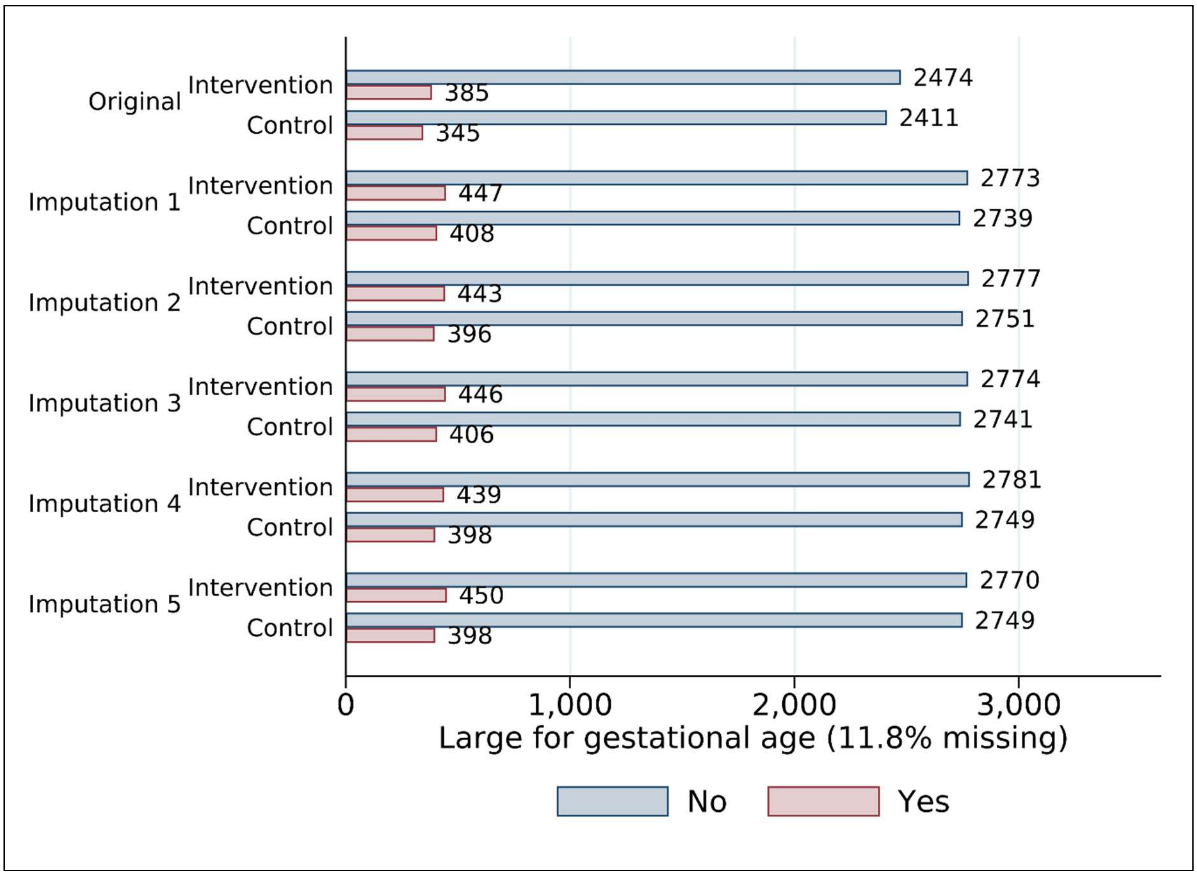
Original
Intervention
Control
Imputation 1
Imputation 2
Imputation 3
Imputation 4
Imputation 5
2474
385
2411
345
2773
447
2739
408
2777
443
2751
396
2774
446
2741
406
2781
439
2749
398
2770
450
2749
398
0
1,000
2,000
3,000
Large for gestational age (11.8% missing)
No
Yes

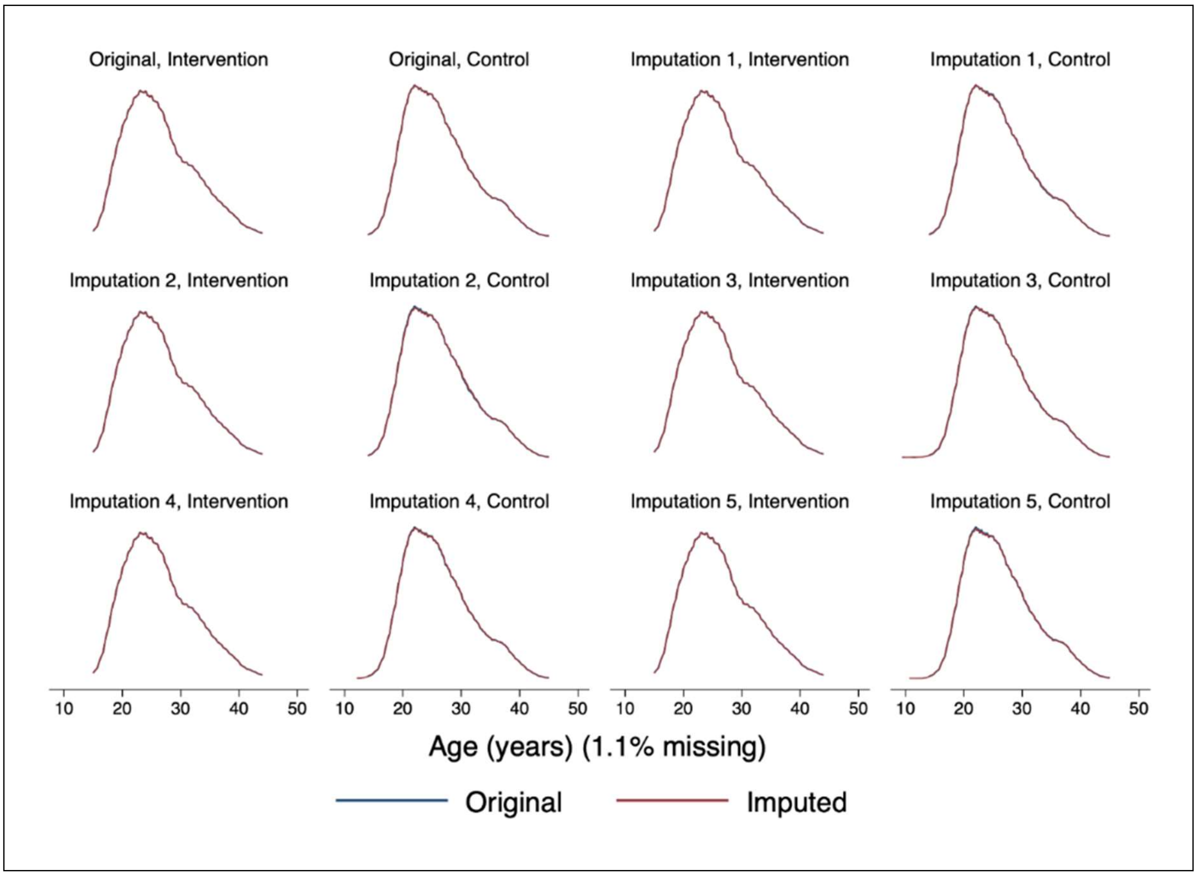
Original, Intervention
Original, Control
Imputation 1, Intervention
Imputation 1, Control
Imputation 2, Intervention
Imputation 2, Control
Imputation 3, Intervention
Imputation 3, Control
Imputation 4, Intervention
Imputation 4, Control
Imputation 5, Intervention
Imputation 5, Control
10 20 30 40 50
Age (years) (1.1% missing)
Original
Imputed

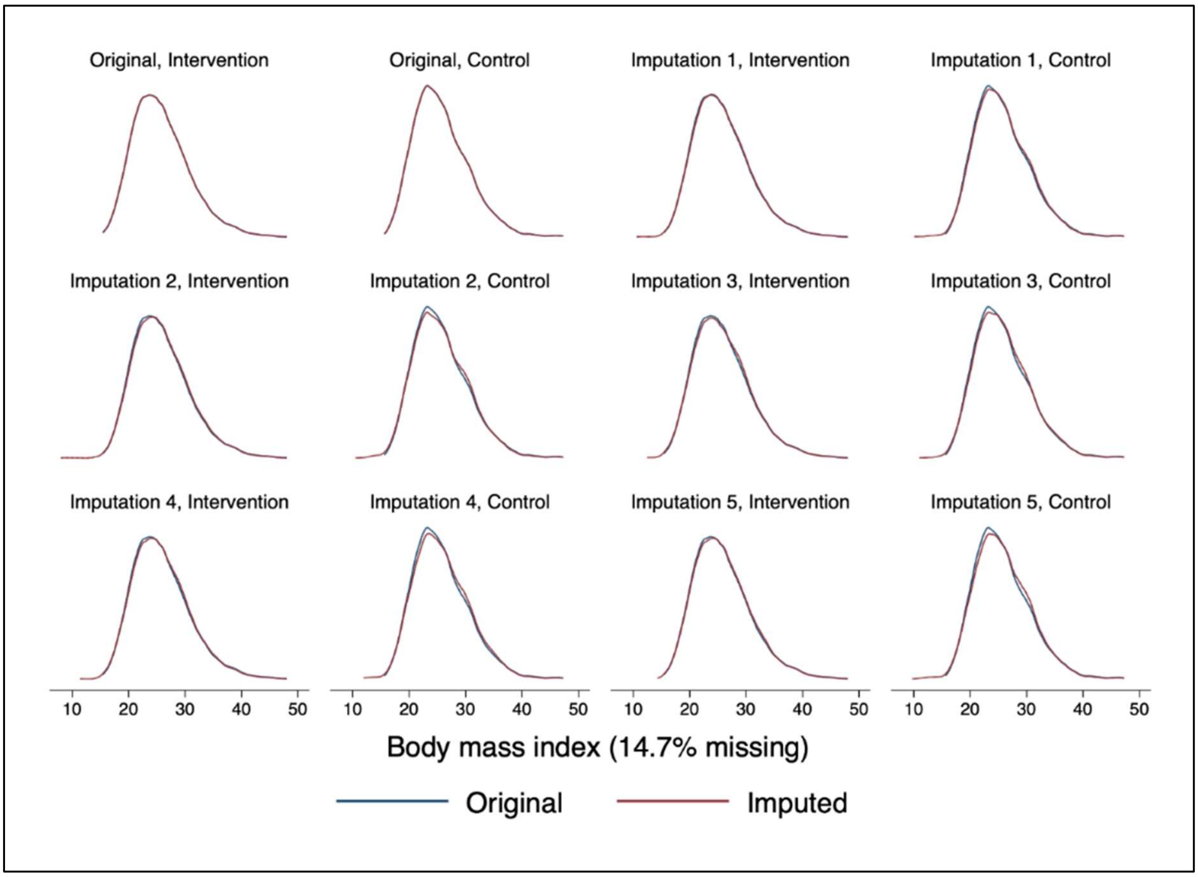
Original, Intervention
Original, Control
Imputation 1, Intervention
Imputation 1, Control
Imputation 2, Intervention
Imputation 2, Control
Imputation 3, Intervention
Imputation 3, Control
Imputation 4, Intervention
Imputation 4, Control
Imputation 5, Intervention
Imputation 5, Control
10 20 30 40 50
Body mass index (14.7% missing)
Original
Imputed

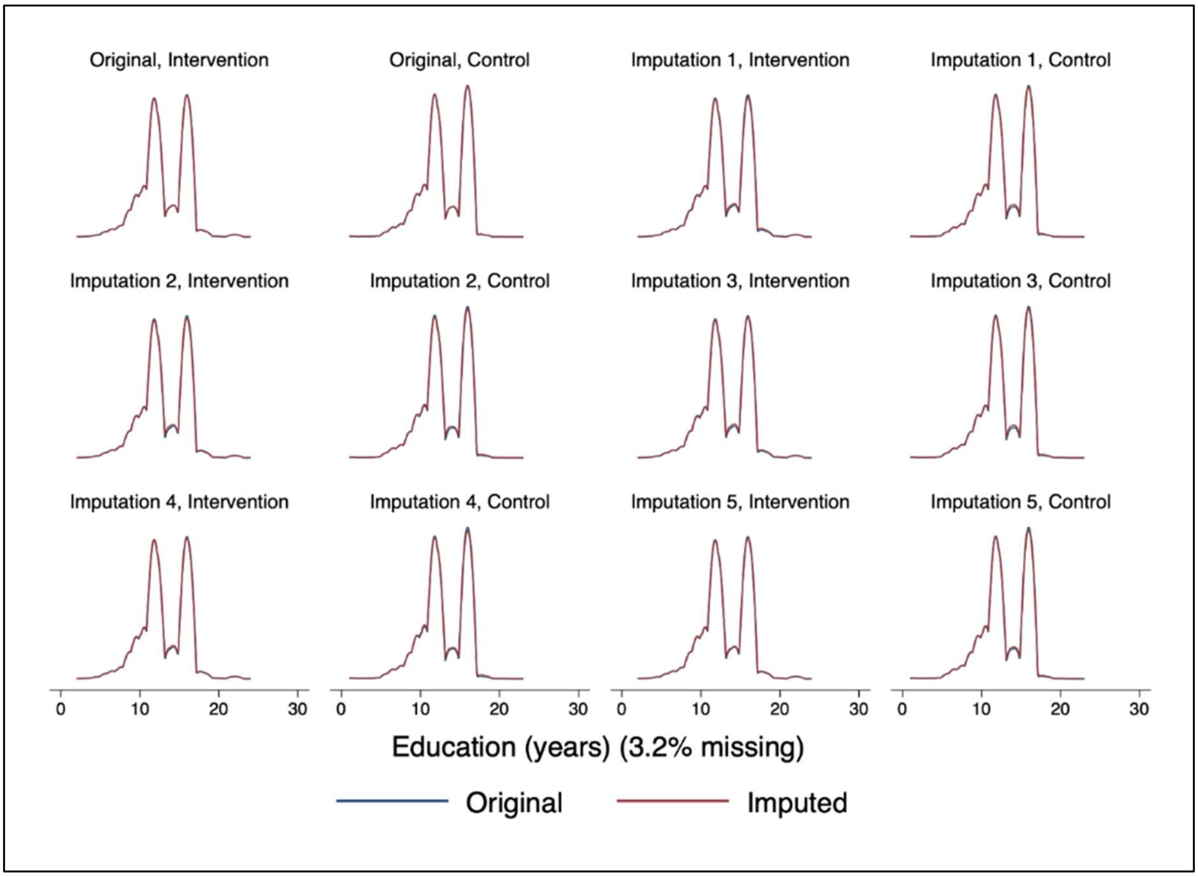
Original, Intervention
Original, Control
Imputation 1, Intervention
Imputation 1, Control
Imputation 2, Intervention
Imputation 2, Control
Imputation 3, Intervention
Imputation 3, Control
Imputation 4, Intervention
Imputation 4, Control
Imputation 5, Intervention
Imputation 5, Control
0 10 20 30
Education (years) (3.2% missing)
Original
Imputed

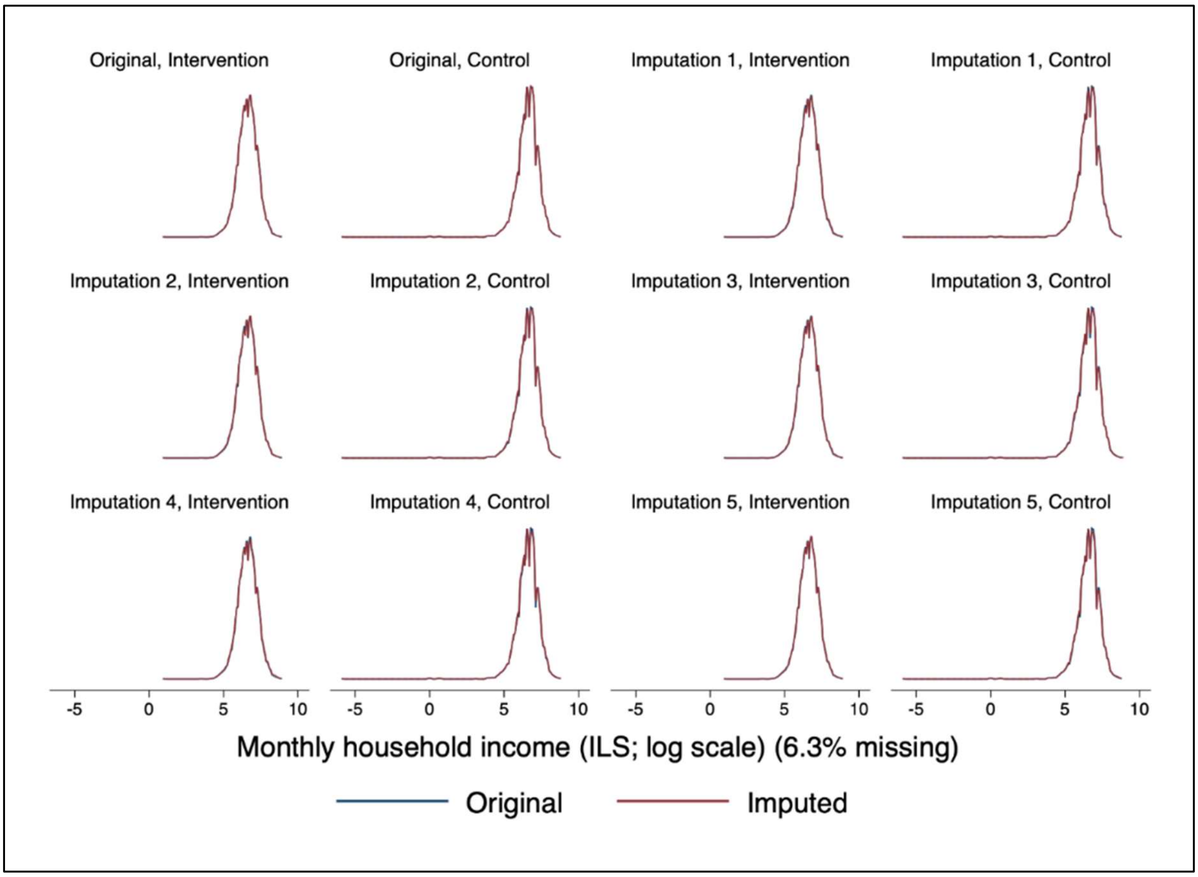
Original, Intervention
Original, Control
Imputation 1, Intervention
Imputation 1, Control
Imputation 2, Intervention
Imputation 2, Control
Imputation 3, Intervention
Imputation 3, Control
Imputation 4, Intervention
Imputation 4, Control
Imputation 5, Intervention
Imputation 5, Control
-5 0 5 10
Monthly household income (ILS; log scale) (6.3% missing)
Original
Imputed

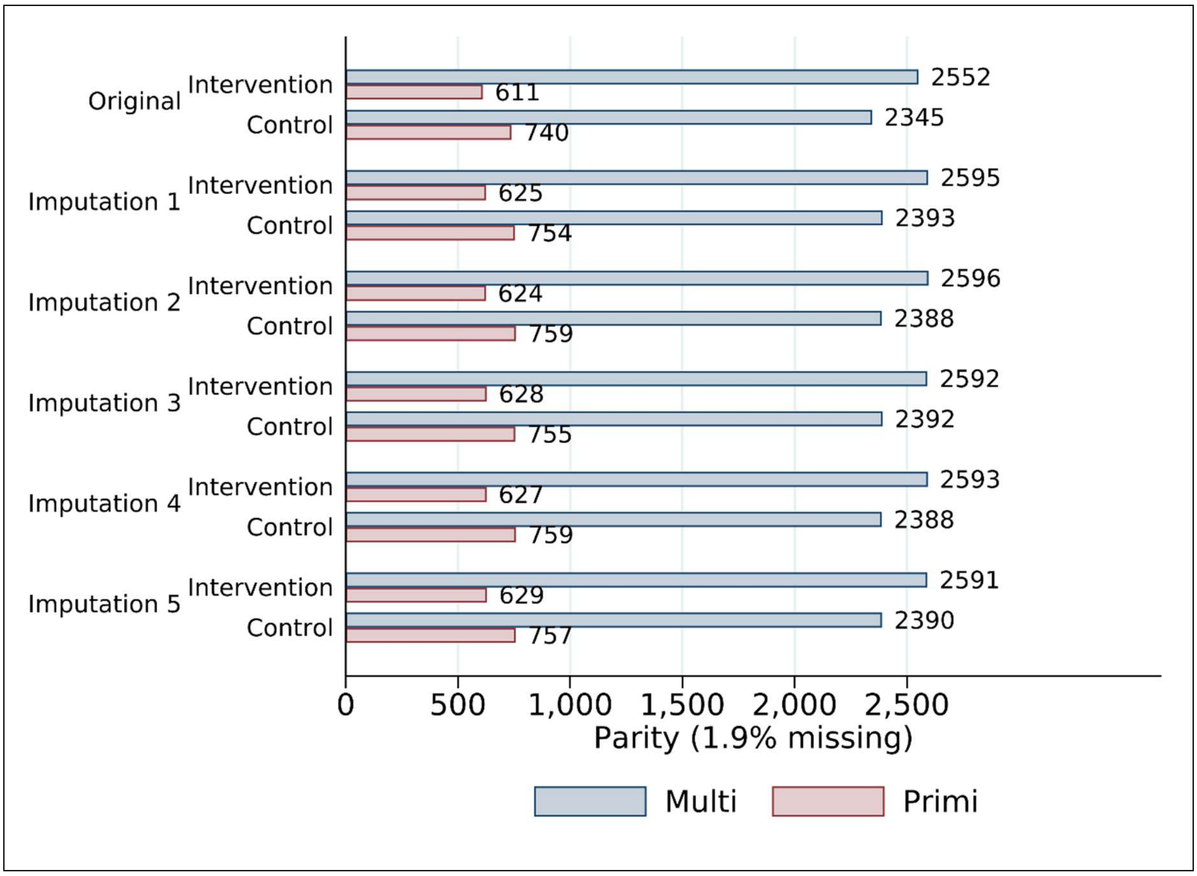

Secondary outcomes

Table. Effect of eRegistry's clinical decision support on secondary outcomes

Secondary outcomes	Control group[§] (N=3148 women)	Intervention group[§] (N=3219 women)	Adjusted odds ratio* (95% CI)
Antenatal care attendance			
Attended an antenatal care visit[¥] given the opportunity	4912/11238 (43·7%)	4502/10475 (42·9%)	0·85 (0·68 to 1·06)
Eligible for a 16-week visit	1738	1572	
Attended a 16-week visit	705 (40·5%)	595 (37·8%)	
Eligible for a 18-22-week visit	2059	1861	
Attended a 18-22-week visit	1161 (56·3 %)	978 (52·5 %)	
Eligible for a 24-28-week visit	2384	2193	
Attended a 24-28-week visit	1134 (47·5 %)	1187 (54·1 %)	
Eligible for a 32-week visit	2519	2403	
Attended a 32-week visit	955 (37·9 %)	897 (37·3 %)	
Eligible for a 36-week visit	2538	2446	
Attended a 36-week visit	957 (37·7 %)	845 (34·5 %)	
Malpresentation			
Screening and management during eligible antenatal contacts	790/1021 (77·5 %)	733/914 (80·2 %)	1·42 (0·92 to 2·19)
Screening during antenatal contacts	778/957 (81·3 %)	726/845 (85·9 %)	
Management of malpresentation	12/63 (19·0 %)	7/41 (17·1 %)	
Stillbirth	20 (6 per 1000)	21 (7 per 1000)	1·07 (0·57 to 2·00)

[§]Unadjusted; *Adjusted for clustering and for repeated antenatal care visits by a woman. [¥]Recommended schedule for routine antenatal care in the West Bank, Palestine is as follows: a first visit before 16 weeks, 16 weeks, 18-22 weeks, 24-28 weeks, 32 weeks, and 36 weeks.

Figure: Plot showing marginal probability of attendance at antenatal care (secondary outcome) with respect to cluster size, age of the woman, laboratory availability and parity

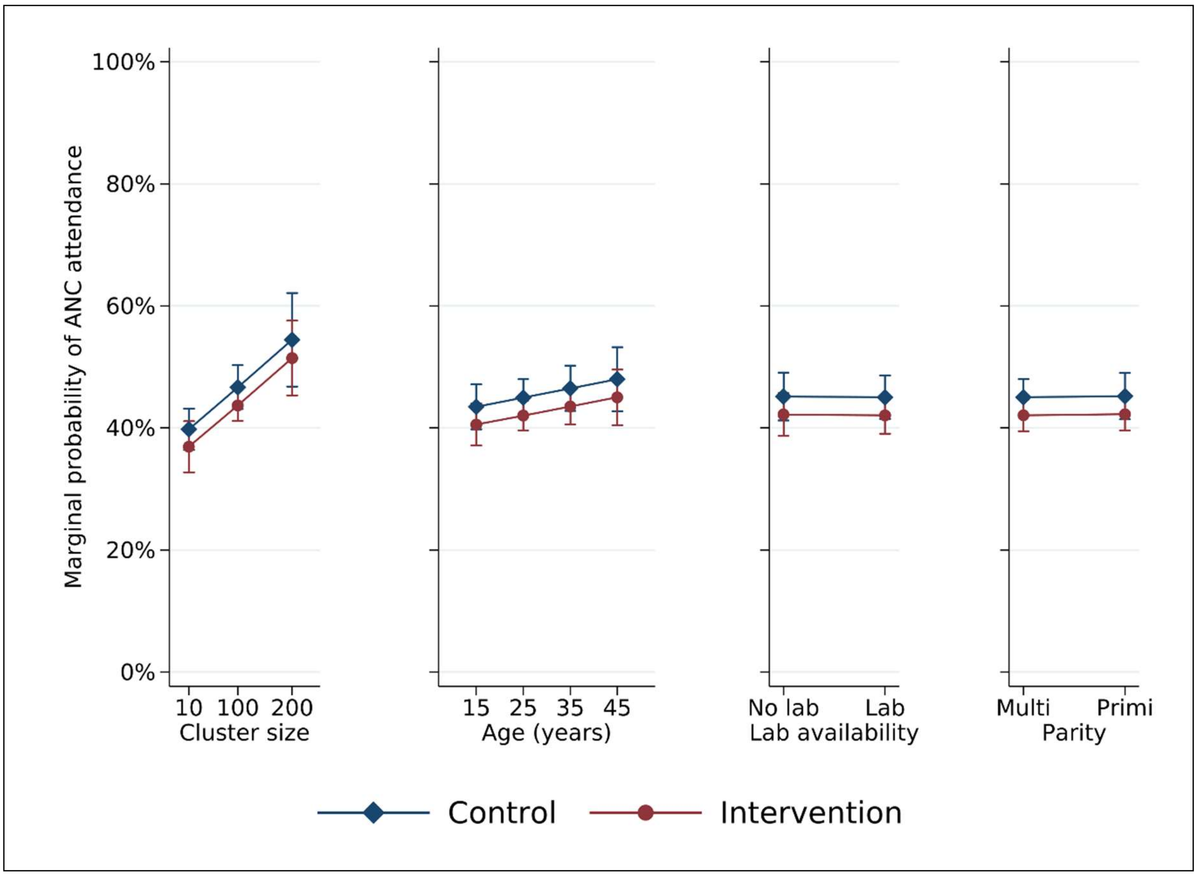

References

1. Hopewell S, Clarke M, Moher D, Wager E, Middleton P, Altman DG, et al. CONSORT for reporting randomised trials in journal and conference abstracts. Lancet 2008, 371:281-283.
2. Hopewell S, Clarke M, Moher D, Wager E, Middleton P, Altman DG at al. (2008) CONSORT for reporting randomized controlled trials in journal and conference abstracts: explanation and elaboration. PLoS Med 5(1): e20.
3. Ioannidis JP, Evans SJ, Gotzsche PC, O'Neill RT, Altman DG, Schulz K, Moher D. Better reporting of harms in randomized trials: an extension of the CONSORT statement. Ann Intern Med 2004; 141(10):781-788.
